# 6.1% Volumetric Negative Thermal Expansion Induced by the Reversible Reconstructive Phase Transition in Pb‐Free Bi_1−_ *_x_Ln_x_* CoO_3_ (*Ln*: Lanthanoid)

**DOI:** 10.1002/advs.77022

**Published:** 2026-09-03

**Authors:** Yuki Sakai, Kazuki Takahashi, Shogo Wakazaki, Takumi Nishikubo, Jun Miyake, Takehiro Koike, Teppei Nagase, Takafumi Yamamoto, Kano Hatayama, Masaichiro Mizumaki, Masaki Azuma

**Affiliations:** ^1^ Kanagawa Institute of Industrial Science and Technology Ebina Kanagawa Japan; ^2^ Materials and Structures Laboratory Institute of Integrated Research, Institute of Science Tokyo Yokohama Kanagawa Japan; ^3^ Neutron Science and Technology Center, Comprehensive Research Organization for Science and Society (CROSS) Ibaraki Japan; ^4^ Research Center for Autonomous Systems Materialogy (ASMat) Institute of Integrated Research Institute of Science Tokyo Yokohama Kanagawa Japan; ^5^ Department of Chemistry, Graduate School of Science Kyoto University Kyoto Japan; ^6^ Faculty of Advanced Science and Technology Kumamoto University Kumamoto Kumamoto Japan; ^7^ Japan Synchrotron Radiation Research Institute (JASRI) Hyogo Japan

**Keywords:** high‐pressure chemistries, negative thermal expansions, perovskite phases, spin crossovers

## Abstract

Negative thermal expansion (NTE) materials, which can compensate for the thermal expansion of structural materials, have attracted much attention in the field of nanoscale electronics and optical devices requiring precise positioning. The present paper demonstrates a reversible colossal NTE in the perovskite‐type oxide lanthanoid‐substituted BiCoO_3._ The 6.1% volume shrinkage in Bi_0.82_Nd_0.18_CoO_3_ is the largest ever observed in Pb‐free NTE materials. Synchrotron X‐ray diffraction and Co L‐edge soft X‐ray absorption spectroscopy measurements and machine learning force field molecular dynamics simulations confirm that the origin of the NTE is the coordination change from the CoO_5_ pyramid with high‐spin Co^3+^ to the CoO_6_ octahedron with the LaCoO_3_‐type Co^3+^ spin state (intermediate‐spin state or mixture of high‐spin and low‐spin states) due to melting of the d*_xy_* orbital ordering in the d^6^ electron configuration. The NTE properties as functions of ionic radius and concentration of substituting *Ln* ions are well explained by the nucleation mechanism of the reconstructive martensitic phase transition. The present results offer a new strategy for developing large‐NTE materials.

## Graphical abstract

Perovskite‐type oxide lanthanoid‐substituted BiCoO_3_ exhibits a reversible negative thermal expansion (NTE) originated from the coordination change from the CoO_5_ pyramid to the CoO_6_ octahedron due to melting of the d*_xy_* orbital ordering in the d^6^ electron configuration, and the 6.1% volume shrinkage in Bi_0.82_Nd_0.18_CoO_3_ is the largest ever observed in the Pb‐free NTE materials.

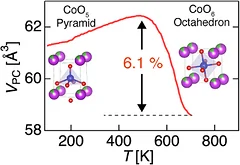

## 1 Introduction

Manufacture of nanoscale electronics and optical devices requires precise positioning, which means that even a small thermal expansion can cause a serious problem. In this situation, materials exhibiting negative thermal expansions (NTEs) have attracted much attention because they can potentially compensate for the thermal expansion of structural materials by forming composites [1, 2, 3, 4, 5, 6, 7, 8, 9, 10]. The last decade has seen remarkable development in materials with NTEs resulting from various volume shrinkage mechanisms. The mechanisms relevant to NTEs are classified into two main groups: open framework‐type and phase transition‐type. The open framework‐type NTEs are driven by anharmonic phonon vibrations. A typical example of these NTE materials is ZrW_2_O_8_ [11]. ZrW_2_O_8_ has a crystal structure of ZrO_6_ octahedron and WO_4_ tetrahedron connected by shared vertices, and there are many voids in the crystal lattice due to the presence of unshared vertices. The NTE in ZrW_2_O_8_ is explained by the rigid unit mode (RUM) model. In this model, a framework structure is viewed as a 3‐D periodic network of interconnecting corner‐sharing polyhedra, which are treated as rigid units. Polyhedral rotations associated with transverse displacements of corner‐sharing atoms consume the voids in the framework, resulting in a volume shrinkage upon heating. However, the RUM model isn't necessarily required for this type of NTE, and materials without RUM such as ZrV_2_O_7_ [12] and Y_2_W_3_O_12_ [13] have been reported to exhibit NTEs. Open framework‐type NTE materials include silicon oxides such as a *β*‐eucryptite (LiAlSiO_4_) [14], ScF_3_ [15, 16] and its related materials such as CaZrF_6_ [17] and ReO_3_ [18], metal cyanides such as Cd(CN)_2_ and Zn(CN)_2_ [19], Prussian blue analogues such as AgB(CN)_4_ and CuB(CN)_4_ [20], metal organic frame‐work (MOF) such as a Cu_3_(btb)_2_ (MOF‐14) [21], etc.,. They tend to show thermal shrinkage over a wide temperature range but have small absolute coefficient of thermal expansion (CTE) values. The phase transition‐type NTEs utilize the abrupt volume change that occurs during a phase transition. Although the operating temperature range is limited, it is possible to achieve a much larger negative linear expansion coefficient that cannot be achieved with the open framework‐type NTE materials. The mechanisms underlying volume contraction during the phase transition are diverse: the magneto volume effect in the magnetic materials, such as anti‐perovskite manganese nitrides Mn_3_AN [22, 23] and La(Fe, Si)_13_‐based compounds [24, 25], charge transfer in SmS‐based monosulfides [26] and LaCu_3_Fe_4_O_12_‐ [27, 28] and BiNiO_3_‐based [29, 30, 31, 32, 33, 34, 35] perovskite‐type oxides, spontaneous ferroelectric polarization in PbTiO_3_‐related perovskite‐type oxides [36, 37, 38, 39, 40, 41], orbital order‐disorder transition in Ca_2_RuO_4_ [42, 43], and Jahn‐Teller and pseudo Jahn–Teller effect [44] in A_2_M_2_O_7_ family such as Fe_2_P_2_O_7_ [45], Cr_2_P_2_O_7_ [46] and Zn_2_P_2_O_7_ [47]. For such phase transition‐type NTE materials, the volume difference between the low‐temperature (LT) and high‐temperature (HT) phases limits the magnitude of NTE, and the transition‐temperature width and negative CTE are in a trade‐off relationship. Therefore, large volume reduction of the parent compound is essential to obtain large NTE compounds. As many precision instruments that are adversely affected by thermal expansion are used near room temperature (RT), it is also important that NTE covers the range of RT. The largest reported negative value of a linear coefficient of thermal expansion (*α_L_*) in commercial NTE materials is −187 ppm/K between 295 and 325 K (in perovskite‐type oxide BiNi_0.85_Fe_0.15_O_3_) [31, 48], the absolute value of which is even larger than that of epoxy resin (*α_L_* = 50–100 ppm/K) [49].

The perovskite‐type oxide PbTiO_3_, a typical ferroelectric material, is an NTE material. PbTiO_3_ has a large tetragonal distortion (*c*/*a* = 1.063) [50] originating from the stereochemical effect of the 6s^2^ lone pair of Pb^2+^, covalent Pb─O bonds, and the second‐order Jahn–Teller effect of Ti^4+^. During the transition from ferroelectric tetragonal (space group *P*4*mm*) to paraelectric cubic (space group *Pm* $\bar{3}$ *m*) phase upon heating, the contraction of the *c*‐axis due to the reduction in the tetragonal distortion exceeds the expansion of the *a*‐axis, leading to a temperature‐induced volume shrinkage [50]. Therefore, materials such as this one with a large *c*/*a* ratio have the potential to display colossal NTE. In particular, the perovskite‐type oxide PbVO_3_ with a largely distorted PbTiO_3_‐type tetragonal structure (*c*/*a* = 1.23) [51] exhibits a colossal NTE when chemically modified. Such a large tetragonal distortion originates from the d*_xy_* orbital ordering of V^4+^ with a 3d^1^ electronic configuration, which is a kind of Jahn–Teller distortion. The stereochemical effect of Pb^2+^ brings one of the oxygen ions in the apex direction closer to V^4+^. As a result, the d^1^ electron of V^4+^ selectively occupies a nondegenerate d*_xy_* orbital for avoiding this oxygen nearby. This d*_xy_* orbital ordering further enhances the polar displacement of ions, leading to a giant tetragonal distortion and coordination change from a VO_6_ octahedron to a VO_5_ pyramid. In addition, PbVO_3_ shows a pressure‐induced phase transition from a polar tetragonal structure with a VO_5_ pyramid to a nonpolar cubic structure with a VO_6_ octahedron due to the melting of the d*_xy_* orbital ordering at 1.8 GPa [52]. The VO_6_ octahedron in the ambient‐pressure tetragonal phase is substantially expanded in the *c*‐direction, enhancing the unit cell volume of the tetragonal phase. A drastic *c*‐axis contraction due to the collapse of the pyramidal coordination induces a large volume shrinkage of 10.6%. We have succeeded in achieving colossal NTE with a volume shrinkage of Δ*V*/*V* = 9.3% between 450 and 700 K, which is the largest value reported for NTE materials, by destabilizing the polar structure through weakening of the stereochemical activity of Pb^2+^ by substituting La^3+^ and Sr^2+^ for Pb^2+^ and by the electron doping V^4+^ by substituting Bi^3+^ for Pb^2+^ [53]. This volume shrinkage is three times as large as that of BiNi_0.85_Fe_0.15_O_3_ (Δ*V*/*V* ∼ 3%). However, materials containing lead are restricted with the exception of the high‐ performance one for which no substitutes exist, such as a lead zirconate titanate (PZT) piezoelectric material. Therefore, lead‐free materials are preferable for practical applications.

The perovskite‐type oxide BiCoO_3_ has a PbTiO_3_‐type tetragonal structure with a giant *c*/*a* ratio of 1.27 [54], which is even larger than that of PbVO_3_. This large tetragonal distortion can also be explained by the d*_xy_* orbital ordering in the d^6^ electron configuration of Co^3+^ with a high‐spin (HS) state, where only the d*_xy_* orbital is doubly occupied. In the case of BiCoO_3_, melting of the d*_xy_* orbital ordering occurs at 3 GPa and is accompanied by a structural transition from a polar tetragonal (space group *P*4*mm*) phase with a CoO_5_ pyramid to a nonpolar orthorhombic (space group *Pbnm*) phase with tilted CoO_6_ octahedra and a spin‐state transition from HS to low‐spin (LS) states as evidenced by the synchrotron X‐ray emission spectroscopy [55]. The pressure‐induced volume shrinkage of BiCoO_3_ (Δ*V*/*V* = 13%) is even larger than that of PbVO_3_. Therefore, lead‐free BiCoO_3_‐based perovskite‐type oxides are expected to show colossal negative thermal expansion exceeding that of PbVO_3_‐based NTE materials. We have carried out electron doping of BiCoO_3_ by substituting Ti^4+^ for Co^3+^ in order to destabilize the tetragonal phase [56]. The decrease of the Co valence state due to the electron doping destabilizes the d*_xy_* orbital ordering, resulting in a composition‐induced tetragonal‐to‐orthorhombic structural phase transition at *x* = 0.25. However, this orthorhombic (space group *Amm*2) structure is different from that of the HP phase of BiCoO_3_, and it irreversibly transformed into a tetragonal phase after annealing at 600 K. Therefore, NTE was not achieved by the electron doping of BiCoO_3_.

In the present work, we developed BiCoO_3_‐based NTE materials by substituting trivalent lanthanoid ions without lone pair activity for Bi^3+^, as was done in the development of PbVO_3_‐based NTE materials. Furthermore, the isovalent substitution is expected to prevent the formation of the undesired phase as observed in BiCo_0.75_Ti_0.25_O_3_. Synchrotron X‐ray diffraction (SXRD), soft X‐ray absorption spectroscopy (XAS), and magnetic susceptibility measurements clarified composition‐induced pyramidal‐to‐octahedral coordination changes accompanied by tetragonal‐to‐orthorhombic phase transitions and spin‐state transitions. Moreover, we achieved reversible NTE utilizing the collapse of the pyramidal coordination due to melting of the d*_xy_* orbital ordering in the d^6^ electron configuration, which has been a long‐desired goal in regard to BiCoO_3_ derivatives, for the first time. We also performed the substitutions of *Ln* ^3+^ other than La^3+^ for Bi^3+^. The study on enhancing NTE through *Ln* ^3+^ substitution was conducted on the charge transfer‐type NTE materials, Bi_1−_ *_x_Ln_x_* NiO_3_, and the width of the temperature hysteresis decreased by decreasing the ionic radius of the substituting *Ln* ^3+^ ions [30]. Therefore, in addition to clarifying the ionic radius dependence of the NTE properties, further improvements in these properties are expected in the BiCoO_3_‐based NTE materials. The NTE properties as functions of ionic radius and concentration of substituting *Ln* ions were found to be well explained by the nucleation mechanism of the reconstructive martensitic phase transition. We also succeeded in reproducing the temperature‐induced phase transition accompanied by NTE by machine learning force field molecular dynamics (MD) simulations and experimentally observed a volume shrinkage of 6.1% in Bi_0.82_Nd_0.18_CoO_3_, which is the largest shrinkage reported so far in Pb‐free NTE materials.

## 2 Results and Discussion

### 2.1 Composition‐Induced Structural Transformations in Bi_1−_ *_x_* La*_x_* CoO_3_

First, we investigated the effect of substituting La^3+^ for Bi^3+^. Figure 1a shows the SXRD patterns of Bi_1−_ *_x_* La*_x_* CoO_3_ (*x* = 0, 0.1, 0.2, 0.3, 0.4, 0.6, 0.8, and 1.0) at RT. With increasing La content, the crystal structure changed from a PbTiO_3_‐type tetragonal one with the *P*4*mm* space group to a GdFeO_3_‐type orthorhombic one with the *Pbnm* space group at *x* > 0.2 and then to a LaCoO_3_‐type rhombohedral one with the *R* $\bar{3}$ *c* space group at *x* > 0.6. Moreover, tetragonal, orthorhombic, and rhombohedral phases coexisted over a wide composition range. Figure 1b shows the composition dependence of the lattice parameters, *c*/*a* ratio, and unit cell volume for the tetragonal phase of Bi_1−_ *_x_* La*_x_* CoO_3_ at RT. The *c*‐axis length decreased with increasing amount of La, while the *a*‐axis length increased, inducing a decrease in the *c*/*a* ratio. The decrease in the *c*/*a* ratio upon La substitution should be attributed to the decrease in the stereochemical 6s^2^ lone‐pair effect of Bi^3+^ because Shannon's crystal ionic radii for eightfold coordinated Bi^3+^ (1.31 Å) and La^3+^ (1.3 Å) are almost the same. Such a decrease in tetragonality by substituting the lone‐pair inactive ion for the active ions has also been observed in PbVO_3_‐ [37, 53, 57] and Bi_0.5_Na_0.5_VO_3_‐based [38, 40] NTE materials. However, the reduction in tetragonal distortion in Bi_1‐_ *_x_* La*_x_* CoO_3_ is not so large in comparison with Pb_1−_ *_x_* Bi*_x_* VO_3_ [37, 53, 57], only 0.70% at *x* = 0.2, and the unit cell volume rather increases with the amount of La. The lattice parameters of Bi_1−_ *_x_* La*_x_* CoO_3_ remained constant for *x* > 0.2, indicating the solubility limit. Figure 1c shows the composition dependence of pseudo cubic volume for tetragonal, orthorhombic, and rhombohedral phases of Bi_1−_ *_x_* La*_x_* CoO_3_ at RT. Although the pseudo cubic lattice volumes of the orthorhombic and rhombohedral phases of Bi_1−_ *_x_* La*_x_* CoO_3_ were almost the same, the composition‐induced tetragonal‐to‐orthorhombic phase transition was accompanied by a colossal volume shrinkage of 14%, which is close to the 13% observed in the pressure‐induced phase transition of BiCoO_3_. Therefore, this volume shrinkage should also be attributed to the coordination change from the CoO_5_ pyramid to the CoO_6_ octahedron due to melting of the d*_xy_* orbital ordering. Figure 1d,e show the Rietveld refinements of the SXRD data for BiCoO_3_ and Bi_0.6_La_0.4_CoO_3_ at RT together with illustrations of the Co coordination environments in the tetragonal (BiCoO_3_) and orthorhombic (Bi_0.6_La_0.4_CoO_3_) phases obtained by the refinements. The refined crystallographic parameters and the reliability factors are summarized in Table S1. The coordination change from the CoO_5_ pyramid to the CoO_6_ octahedron is confirmed, and the bond valence sum value of 2.98 for the Co ion in the orthorhombic phase indicates the absence of a Co valence change from trivalent. The crystal ionic radius of Co^3+^ in the orthorhombic phase estimated by subtracting the oxide radius of 1.26 Å from the average Co─O bond length is 0.701(4) Å. As Shannon's ionic radii of sixfold coordinated Co^3+^ for LS and HS states are 0.685 and 0.75 Å, respectively, this coordination change seems to be accompanied by a spin‐state transition from the HS state.

**FIGURE 1:**
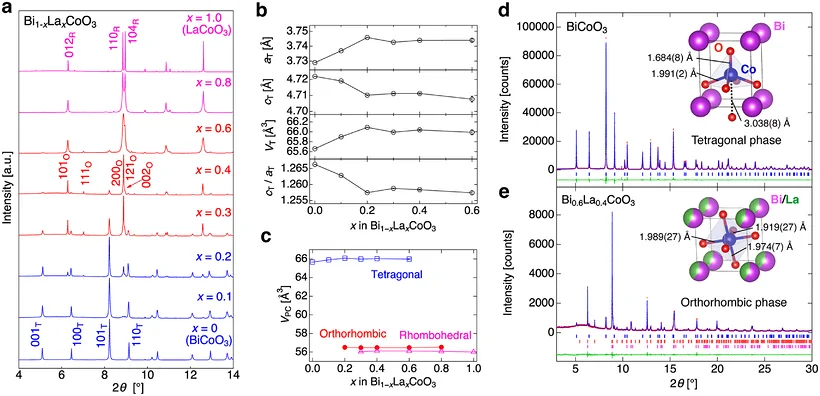
SXRD patterns indicating a composition‐induced structural transition from a polar tetragonal phase with a CoO_5_ pyramid to a nonpolar orthorhombic phase with tilted CoO_6_ octahedra accompanied by a colossal volume shrinkage of 14%. (a) SXRD patterns of Bi_1‐_ *_x_* La*_x_* CoO_3_ at RT. The wavelength used is 0.420264 Å. The labels attached to the reflection indices (T, O, and R) indicate reflections from tetragonal, orthorhombic, and rhombohedral phases, respectively. (b) Composition dependence of lattice parameters, unit cell volume, and *c*/*a* ratio for the tetragonal phase of Bi_1−_ *_x_* La*_x_* CoO_3_ at RT. (c) Composition dependence of pseudo‐cubic volumes for tetragonal, orthorhombic, and rhombohedral phases of Bi_1−_ *_x_* La*_x_* CoO_3_ at RT. (d,e) Observed (red points), calculated (blue line), and difference (green line) patterns from the Rietveld analysis of the SXRD data of BiCoO_3_ (d) and Bi_0.6_La_0.4_CoO_3_ (e) at RT with illustrations of the Co coordination environments in the tetragonal (BiCoO_3_) and orthorhombic (Bi_0.6_La_0.4_CoO_3_) phases obtained by the refinements. The tick marks correspond to the positions of Bragg reflections of the *P*4*mm* tetragonal phases (blue), *Pnma* orthorhombic phases (red), and *R* $\bar{3}$ *c* rhombohedral phases (purple).

Co L‐edge XAS spectroscopy was performed in order to confirm this transition directly. Figure 2a shows the XAS spectra at RT obtained using the inverse partial fluorescence yield (IPFY) method, and Figure S1 summarizes those obtained by the total electron yield (TEY) and partial fluorescence yield (PFY) methods. TEY is a surface‐sensitive method, and the PFY spectra are distorted by the self‐absorption effect. Thus, we evaluated the spin state by using the IPFY method. This method is both bulk‐sensitive and free from the self‐absorption effect, and the data for it were obtained from the inverse of the oxygen PFY spectra in the Co L‐edges region [58]. The peak energy and shape of the Bi_0.6_La_0.4_CoO_3_ spectra are more similar to those of LaCoO_3_ than to those of BiCoO_3_. There is an ongoing debate as to whether the Co^3+^ spin state of LaCoO_3_ at RT is an intermediate‐spin state (IS) or a mixed spin state of HS (octahedral coordination) and LS, but we will not discuss it in the present paper. In any case, however, the spin state of LaCoO_3_ at RT is not completely the HS state. The Co^3+^ spin state of the orthorhombic phase was also evaluated from the magnetic property of Bi_0.6_La_0.4_CoO_3_. Figure 2b,c show the temperature dependence of the magnetic susceptibility measured on heating after zero‐field cooling (ZFC) and on cooling in the field (FC), and the magnetization curves at various temperatures for Bi_0.6_La_0.4_CoO_3_, respectively. Bi_0.6_La_0.4_CoO_3_ is a canted antiferromagnet with a Néel temperature of 15 K and a remanent magnetization of 0.0048 *μ*_B_ /Co at 5 K, indicating that Co^3+^ is not completely nonmagnetic state even at LT. The Néel temperature far below that of BiCoO_3_ (470 K) [54] suggests the spin state change. The magnetic susceptibility data between 100 and 400 K, well above the Néel temperature, can be fitted to the Curie‐Weiss law with a temperature‐independent term *χ*_0_, *χ* = *C*/(*T* − *θ*) + *χ*_0_. Here, *C* is the Curie constant, and *θ* is the Weiss temperature. The fitting gives *χ*_0_ = 8.7(2) × 10^−4^ emu/mol, *C* = 0.500(8) emu·K/mol, and *θ* = −5(2) K. The effective magnetic moments calculated from the Curie constant was 2.00 *μ*_B_/Co, which is far from the expected values for LS state with *S* = 0 (0 *μ*_B_) and HS state with *S* = 2 (4.90 *μ*_B_), but is close to 2.83 *μ*_B_ expected for IS state with *S* = 1. It should be noted that the HS in BiCoO_3_ has been established by the ordered magnetic moment of 3.24 *μ*_B_ [54]. Therefore, the comprehensive studies of SXRD, XAS, and magnetic measurements confirm the composition‐induced phase transition from the polar tetragonal phase with Co^3+^ high‐spin state in the CoO_5_ pyramidal coordination to the nonpolar orthorhombic phase with the LaCoO_3_‐type Co^3+^ spin state in the CoO_6_ octahedral coordination, as we expected.

**FIGURE 2:**
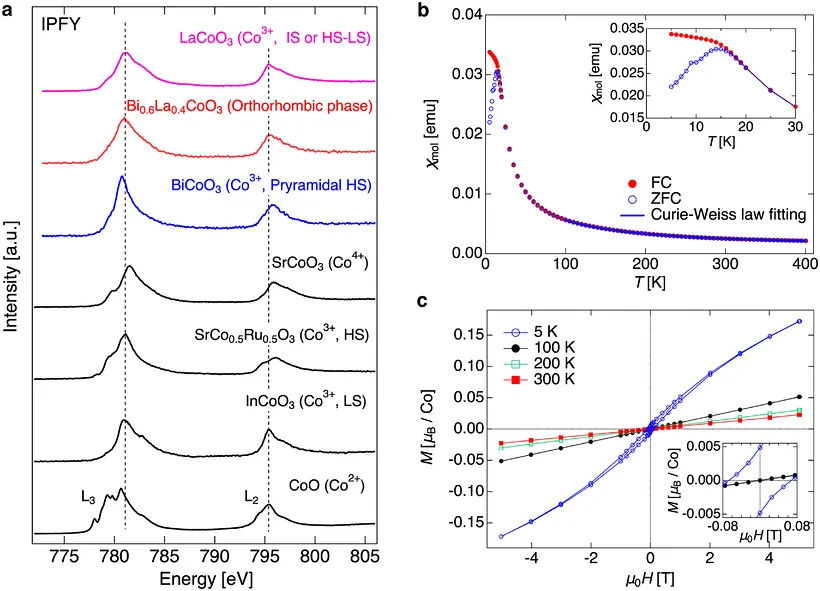
XAS spectroscopy and magnetic measurements confirming the spin state transition from HS to the LaCoO_3_‐type Co^3+^ spin state induced by the composition‐induced tetragonal‐to‐orthorhombic phase transition. (a) Co L‐edge IPFY XAS spectra of Bi_0.6_La_0.4_CoO_3_ at RT together with those of CoO, InCoO_3_ [59], SrCo_0.5_Ru_0.5_O_3_ [60], LaCoO_3_, SrCoO_3_, and BiCoO_3_ as references of Co^2+^, Co^3+^ (LS), Co^3+^ (HS), Co^3+^ (IS or the HS‐LS mixed spin state), and Co^4+^ in octahedral coordinations and HS Co^3+^ in a pyramidal coordination, respectively. The dotted lines indicate the peak positions of the LaCoO_3_ spectra. (b) Temperature dependence of molar magnetic susceptibility for Bi_0.6_La_0.4_CoO_3_. The inset is a magnified view near the Néel temperature. (c) The magnetization curves of Bi_0.6_La_0.4_CoO_3_ at various temperatures. The inset is a magnified view near zero magnetic field.

### 2.2 Temperature‐Induced Structural Transformations and NTE Properties in Bi_1−_ *_x_* La*_x_* CoO_3_

If this tetragonal‐to‐orthorhombic phase transition occurs upon heating, there should be a large NTE. Thus, we investigated the temperature‐induced structural change in La‐substituted compounds. Figure 3a shows the SXRD patterns for Bi_0.8_La_0.2_CoO_3_ at various temperatures upon heating and cooling. As the sample gradually decomposed above 700 K (Figure S2), the maximum temperature was set below 700 K. The peak intensities derived from the orthorhombic phase increased upon heating, while those from the tetragonal phase conversely decreased. Moreover, the peak intensities returned to the original values on cooling. These results indicate a reversible temperature‐induced tetragonal‐to‐orthorhombic phase transition. Figure 3b shows the temperature dependence of the pseudo‐cubic volume of the tetragonal (*V*_PC(T)_) and orthorhombic (*V*_PC(O)_) phases, fraction of orthorhombic phase (*f*_O_), and weighted average volume, *V*_ave_ = *f*_O_ × *V*_PC(O)_ + (100 − *f*_O_) × *V*_PC(T)_, for Bi_0.8_La_0.2_CoO_3_. Although the lattice parameters of the tetragonal and orthorhombic phases monotonically increased, the fraction of HT orthorhombic phase increased upon heating, resulting in a negative thermal expansion with *α_L_* = −46.6 ppm/K between 550 and 700 K, as approximated by *α_L_* = 1/3*α_V_*. Figure 3c shows the temperature dependence of the weighted average volume upon heating for samples with various La concentrations near the tetragonal and orthorhombic phase boundary. Figure S3 shows SXRD patterns at minimum and maximum temperatures upon heating and cooling, and Figure S4 plots the temperature dependence of the pseudo‐cubic volume of the tetragonal and orthorhombic phases and the fraction of the orthorhombic phase. The samples with *x* = 0.18 and 0.21 also showed NTE, but the *x* = 0.20 sample showed the largest volume shrinkage. Moreover, the transition temperatures showed almost no composition dependence.

**FIGURE 3:**
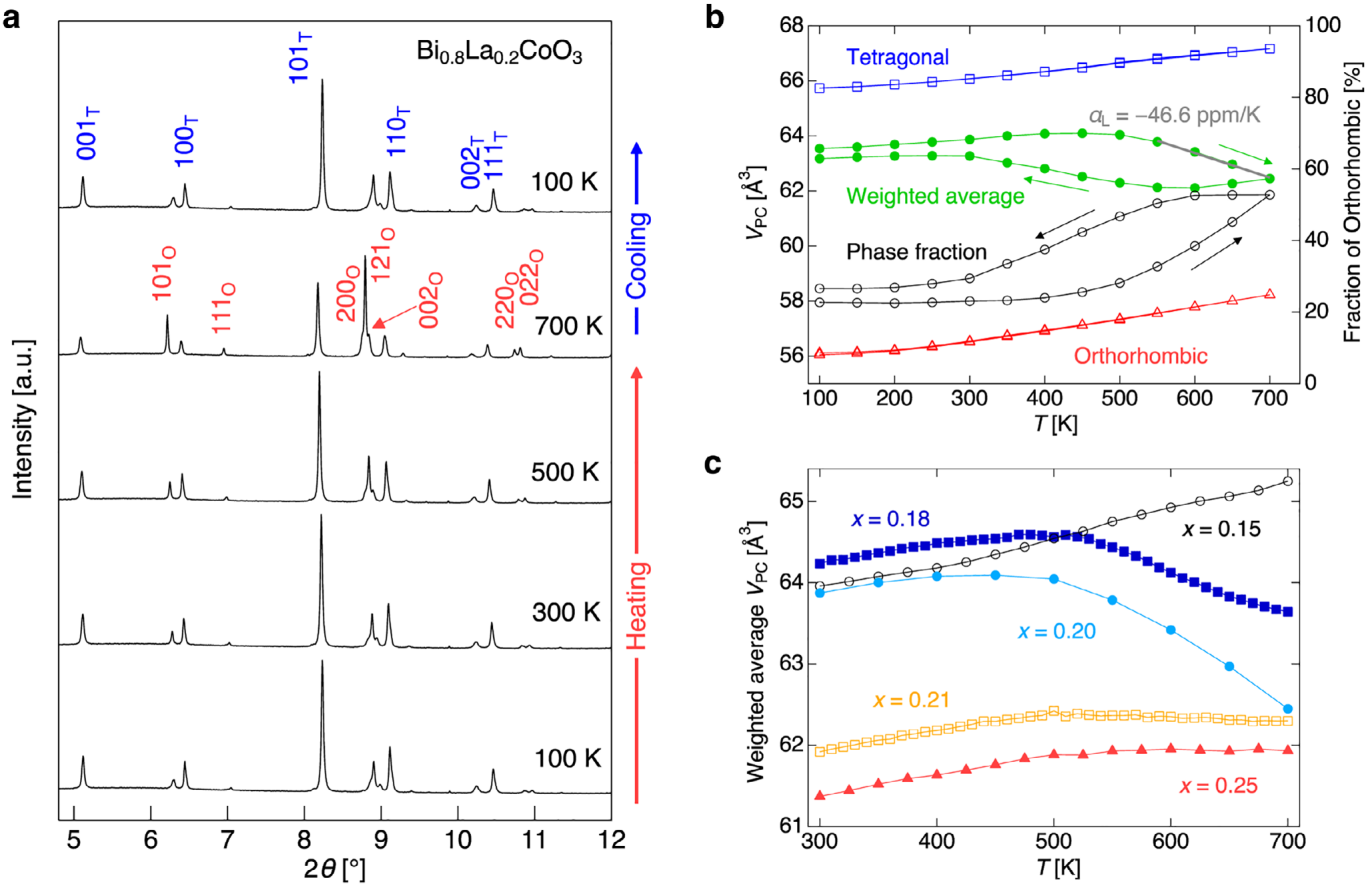
Temperature dependence of weighted average volume calculated from the SXRD patterns confirming the temperature‐induced tetragonal‐to‐orthorhombic phase transition accompanied by NTE. (a) SXRD patterns of Bi_0.8_La_0.2_CoO_3_ at various temperatures upon heating and cooling. The wavelength used is 0.420264 Å. The labels attached to the reflection indices (T and O) indicate reflections from tetragonal and orthorhombic phases, respectively. (b) Temperature dependence of pseudo cubic volume of tetragonal and orthorhombic phases, fraction of the orthorhombic phase, and weighted average volume for Bi_0.8_La_0.2_CoO_3_. *α_L_* = −46.6 ppm/K was obtained by a linear approximation fitting of the weighted average volume in the temperature range depicted as a gray bold line. (c) Temperature dependence of weighted average pseudo cubic volume for Bi_1−_ *_x_* La*_x_* CoO_3_.

The volume shrinkage of 2.6% observed in the *x* = 0.20 sample was much smaller than that expected from the difference in volume between the tetragonal and orthorhombic phases (Δ*V*/*V* ∼ 14% at 600 K) because the transition was not complete and the fraction of the orthorhombic phase increased only by 30%, from 23% to 53%, below the decomposition temperature. The time‐lapse SXRD patterns of Bi_0.8_La_0.2_CoO_3_ at 650 K (Figure S5) indicate that these transitions are almost of athermal, i.e., the proceeding of the transition depends only on temperature and not on holding time. The athermal nature is often observed in transitions accompanied by large strains, such as the martensitic transition of ZrO_2_ [61] and transitions of phase‐transition‐type NTE materials such as PbVO_3_ derivatives [53]. Phase stabilities in the martensitic transitions are influenced not only by the Gibbs free energy, but strain and interfacial free energies have large influences. The thermal fluctuation energy is insufficient to overcome the latter energies, and the driving force of the transition is the increase in the difference of the Gibbs free energies between the initial and final phases, which is the origin of the athermal nature [62, 63]. The athermal transitions in NTE materials would also be caused by the large strain and the interfacial free energies. The domain boundary between the tetragonal and cubic phases in the PbVO_3_ derivatives were investigated by high‐angle annular dark‐field scanning transmission electron microscopy (HAADF‐STEM) and Bragg coherent X‐ray diffraction imaging for Pb_0.82_Sr_0.18_VO_3_ with 68% tetragonal and 32% cubic phases [53]. The {101}‐type partially coherent interface between tetragonal and cubic phases expected for PbTiO_3_‐type ferroelectrics was observed, and large inner strain with the effective pressure of 0.2 GPa was confirmed from the comparison of the interatomic distance at the interface with the values under pressure. These results indicate that, in order to clarify the phase transition behavior during the transition accompanied by a volume shrinkage exceeding 10%, it is necessary to consider the effects of domain structure and lattice mismatch between the phases, as in the case of the martensitic transformation.

### 2.3 Temperature‐Induced Phase Transition Reproduced by MD Simulations

The experimentally observed temperature‐induced phase transition accompanied by NTE was successfully reproduced by molecular dynamics (MD) simulations based on the Crystal Hamiltonian Graph neural Network (CHGNet) machine learning force field [64]. 4 × 4 × 4 pseudocubic unit cells were employed, and the symmetry was lowered to *P*1 as indicated in the cif file found in the Supporting Information in order to reproduce the structural transition from *P*4*mm* to *Pbnm* phases and the random distribution of substituted elements. First, we evaluated the performances of the present MD simulations by analyzing the pressure‐induced phase transition. Figure 4a shows the simulated pressure dependence of the pseudo‐cubic lattice parameters and lattice volume for BiCoO_3_ at 10 K. The simulation results show a large volume shrinkage accompanied by a drastic reduction in the *c*‐axis length, leading to the coordination change from the CoO_5_ pyramid to the CoO_6_ octahedron (Figure S6 and Movie S1) at 3.5 GPa, indicating that the present simulations well reproduce the experimental results. Next, we investigated the temperature‐induced phase transition of La substituted BiCoO_3_. Figure 4b shows the simulated temperature dependence of the pseudo‐cubic lattice parameters and lattice volume for Bi_0.82_La_0.18_CoO_3_. Bi_0.82_La_0.18_CoO_3_ shows a temperature‐induced colossal volume shrinkage accompanied by the drastic contraction in *c*‐axis and the coordination change from the CoO_5_ pyramid to the CoO_6_ octahedron as well as the simulation data of BiCoO_3_ under pressures (Figure 4c and Movie S2). The transition completes within 8 K, which is different from experimentally observed coexistence of tetragonal and orthorhombic phases in the entire temperature range. The present MD simulation considers single domain structure and ignores the grain boundaries, defects, domain size, and polycrystalline orientation distributions, resulting in the discrepancy between simulation and experiment. The simulation result for BiCoO_3_ upon heating showed no structural phase transition below the decomposition temperature (Figure S7), theoretically confirming the necessity of La‐substitution for the appearance of NTE.

**FIGURE 4:**
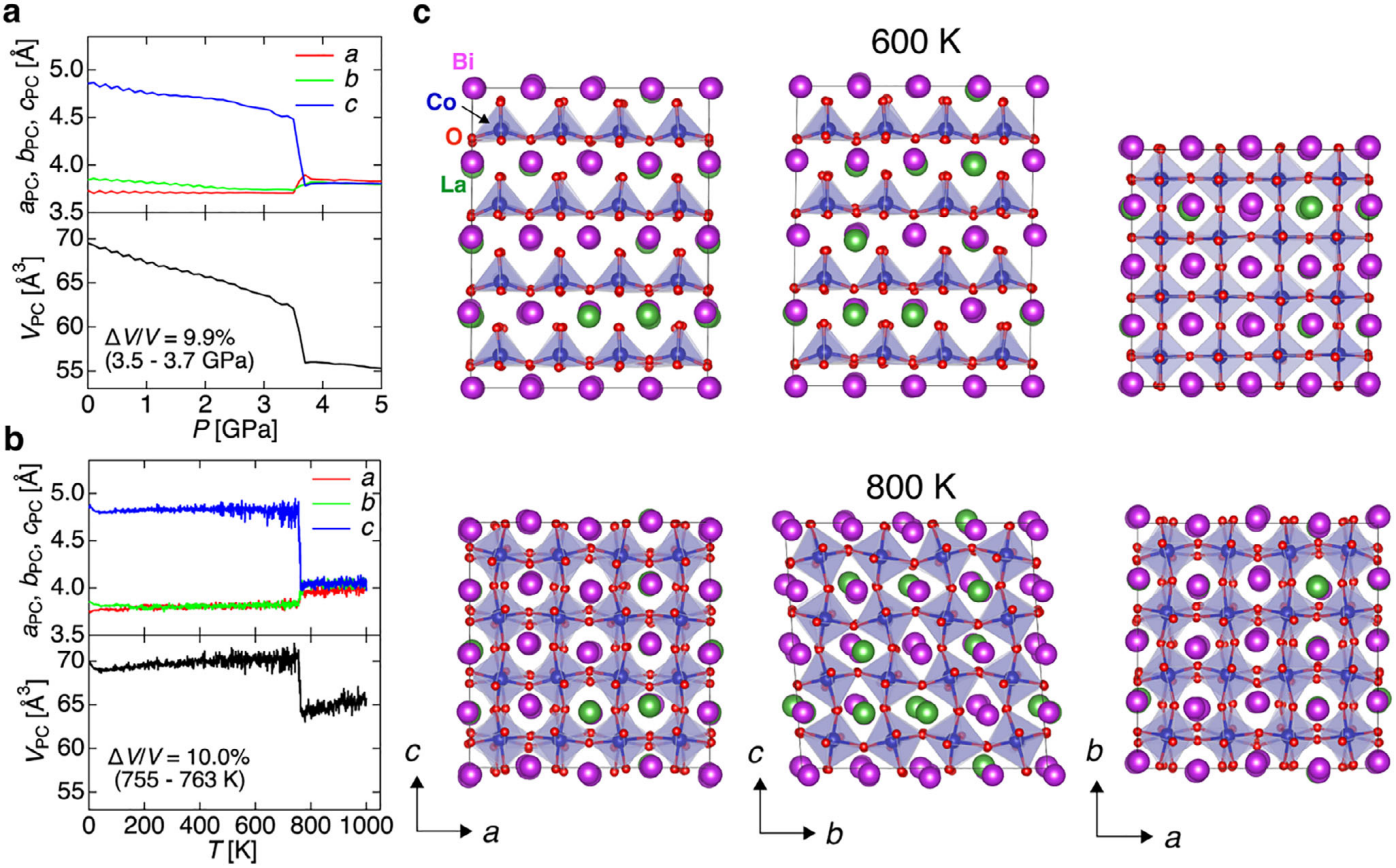
MD simulations reproducing NTE accompanied by coordination change in Bi_0.82_La_0.18_CoO_3_. (a) Simulated pressure dependence of the pseudo‐cubic lattice parameters and pseudo‐cubic unit cell volume for BiCoO_3_. (b) Simulated temperature dependence of the pseudo‐cubic lattice parameters and pseudo‐cubic unit cell volume for Bi_0.82_La_0.18_CoO_3_. (c) Crystal structures of Bi_0.82_La_0.18_CoO_3_ reproduced by MD simulations at 600 and 800 K. These structures were obtained by averaging the lattice parameters and atomic coordinates over data within 5 K to suppress the effect of thermal oscillation.

### 2.4 Optimizing *Ln*‐Substitution Effects for Structural Distortions and NTE Properties

The incomplete temperature‐induced phase transition weakened the volumetric NTEs in Bi_1−_ *_x_* La*_x_* CoO_3_. In other words, an even larger NTE should be achieved by increasing the magnitude of the phase‐fraction change. The ionic radii of the lanthanoid ions range from 1.3 Å (La^3+^) to 1.206 Å (Eu^3+^). Some lanthanoid elements have charge degrees of freedom, e.g., Pr has +3.67 oxidations state in Pr_6_O_11_. Moreover, LaCoO_3_ has a rhombohedral structure, while other *Ln*CoO_3_ have the same orthorhombic structure as the HP phase of BiCoO_3_. These different characteristics of lanthanoids other than La may increase the phase‐fraction change in temperature‐induced phase transitions. Thus, we decided to investigate the crystal structure and NTE behavior of compounds with various lanthanoid‐ion substitutions. Figure 5a shows the SXRD patterns of Bi_0.8_ *Ln*_0.2_CoO_3_ (*Ln* = La, Pr, Nd, Sm, and Eu) at RT. The tetragonal and orthorhombic phases coexisted in all of the compounds, and the phase fraction of the orthorhombic phase systematically increased with increasing concentration and decreasing ionic radius of lanthanoid ions (Figure 5b). The stabilization of the orthorhombic phase is consistent with the tendency of Goldschmidt's tolerance factor whereby smaller A‐site ions make the orthorhombic structure more stable [65, 66].

**FIGURE 5:**
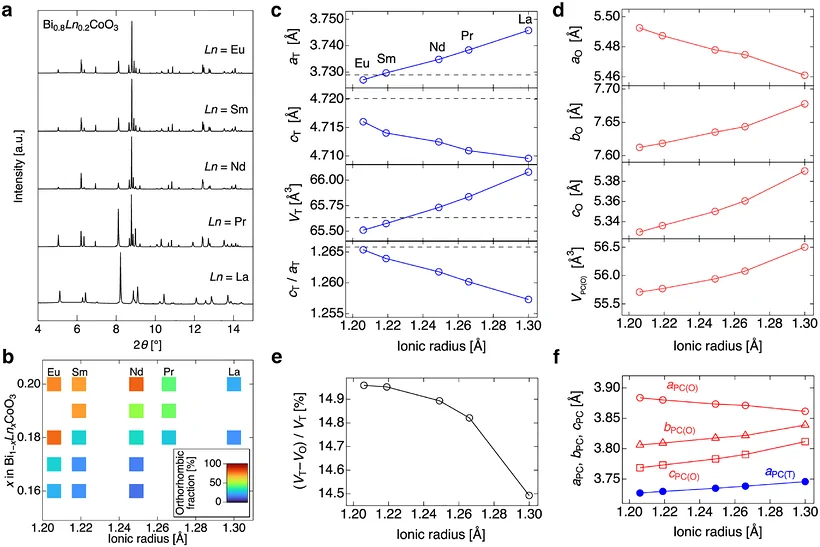
Ionic radius dependence of lattice parameters calculated from the SXRD patterns of Bi_0.8_ *Ln*_0.2_CoO_3,_ indicating that the distortions in both tetragonal and orthorhombic phases vary sufficiently to affect their phase stabilities and lattice mismatch between them depending on the substituting elements. (a) SXRD patterns of Bi_0.8_ *Ln*_0.2_CoO_3_ at RT. The wavelengths used are 0.420264 Å for *Ln* = La and 0.413894 Å for *Ln* ≠ La. (b) Fraction of orthorhombic phase in Bi_1−_ *_x_Ln_x_* CoO_3_ as a function of ionic radius and concentration of substituting *Ln* ions. (c–f) Ionic radius dependence of lattice parameters, unit cell volume, and *c*/*a* ratio of the tetragonal phase of Bi_0.8_ *Ln*_0.2_CoO_3_ at RT (c), lattice parameters and unit cell volume of the orthorhombic phase (d), volume difference between tetragonal and orthorhombic phases (e), and pseudo‐cubic lattice parameters of tetragonal and orthorhombic phases (f). The horizontal dotted lines in (c) indicate the lattice parameters and *c*/*a* ratio of BiCoO_3_.

Figure 5c shows the dependence of the lattice parameters, lattice volume, and *c*/*a* ratio on lanthanoid ionic radius for the tetragonal phase of Bi_0.8_ *Ln*_0.2_CoO_3_ in comparison with those of BiCoO_3_ (dashed lines). The monotonic changes in the lattice parameters with ionic radius indicate that all lanthanoid ions were substituted in a trivalent state. The La substitution decreased the *c*/*a* ratio the most, whereas the other *Ln* ions induced smaller changes as their radii decreased. These results indicate that lanthanoid ions with a smaller ionic radius are less effective in suppressing the stereochemical activity of lone‐pair electrons. Although the contraction in the *c*‐axis due to the weakening of the stereochemical activity is accompanied by the expansion in the *a*‐axis to maintain the bond lengths, the decrease of the lattice volume through the substitution of small lanthanoid ions induces the contraction in the *a*‐axis. Therefore, the contraction in the *c*‐axis is suppressed to avoid excessive shortening of the bond lengths. The contraction in the *a*‐axis and the expansion in the *c*‐axis with decreasing the ionic radius of the substituting ions have also been reported for a comparison of Pb_0.8_Ca_0.2_TiO_3_ and Pb_0.8_Sr_0.2_TiO_3_ [67, 68]. Figure 5d plots the lattice parameters and lattice volumes for the orthorhombic phase of Bi_0.8_ *Ln*_0.2_CoO_3_ as a function of lanthanoid ionic radius. The monotonic changes in the lattice parameters indicate that Co spin states in the orthorhombic phases are the same as in La‐substituted compounds and that lanthanoid ions are substituted in a trivalent state as well as tetragonal phase. The increased orthorhombic distortion (the *b*‐ and *c*‐axis decrease in length while the *a*‐axis increases) with decreasing ionic radius of lanthanoid ions is in accordance with the decreased tolerance factor as discussed above. Figure 5e plots the volume difference between the tetragonal and orthorhombic phases as a function of lanthanoid ionic radius. The large volume difference in the La‐substituted compound slightly increases with decreasing ionic radius, which is mainly caused by the increased orthorhombic distortion (Figure 5f). These results indicate that the distortions in the tetragonal and orthorhombic phases vary sufficiently to affect their phase stabilities and lattice mismatch between them depending on the substituting elements.

As mentioned in the previous section, the tetragonal‐to‐orthorhombic phase transition is strongly affected by domain structure and lattice mismatch between the phases, as in the case of the martensitic transformation. Figure 6a,b show the schematic images of the relationship between the structural distortions, the free energies, and the critical temperatures in martensitic transitions. *G*_T_ *, G*_O_, Δ*G* (= |*G*T – *G*O|), *G*_NC_, *T*_On(H)_, *T*_On(C)_, *T*_Off(C)_, *T*_Hys_ (= *T*_On(H)_ – *T*_Off(C)_), *T*_E_ (= (*T*_On(H)_ + *T*_On(C)_)/2), *T*_Max_, and Δ*f*_O_ indicate the Gibbs free energies of the tetragonal and orthorhombic phases, the relative Gibbs free energy, the non‐chemical free energy, the onset temperature of the transition in the heating process, the onset and offset temperatures in the cooling process, the width of thermal hysteresis, the equilibrium temperature, and the maximum temperature without decomposition, and the fraction change of the orthorhombic phase, respectively. The martensitic transformation starts when the relative Gibbs free energy overcomes the non‐chemical free energy, including strain and interfacial energies (*T*_On_) [69], indicating that a larger difference in the Gibbs free energy (Δ*G*) and a smaller lattice mismatch between LT and HT phases leading to smaller *G*_NC_ are advantageous for increasing the phase‐fraction change. The magnitudes of the tetragonal and orthorhombic distortions increase with decreasing ionic radius of substituting *Ln* ions, resulting in the increase of the lattice mismatch and *G*_NC_. The free energies of both tetragonal and orthorhombic phases at RT decrease with decreasing ionic radius of A‐site, and the increase of the orthorhombic phase fraction at RT with decreasing the ionic radius (Figure 5b) indicates that the latter energy decreases faster. The relative energy lowering of the orthorhombic phase induces the increase of Δ*G* at *T*_Max_. Therefore, both *G*_NC_ and Δ*G* increase with decreasing the ionic radius, which means that the improvement of the NTE properties depends on the order of ionic radius dependence, *G*_NC_ and Δ*G*.

**FIGURE 6:**
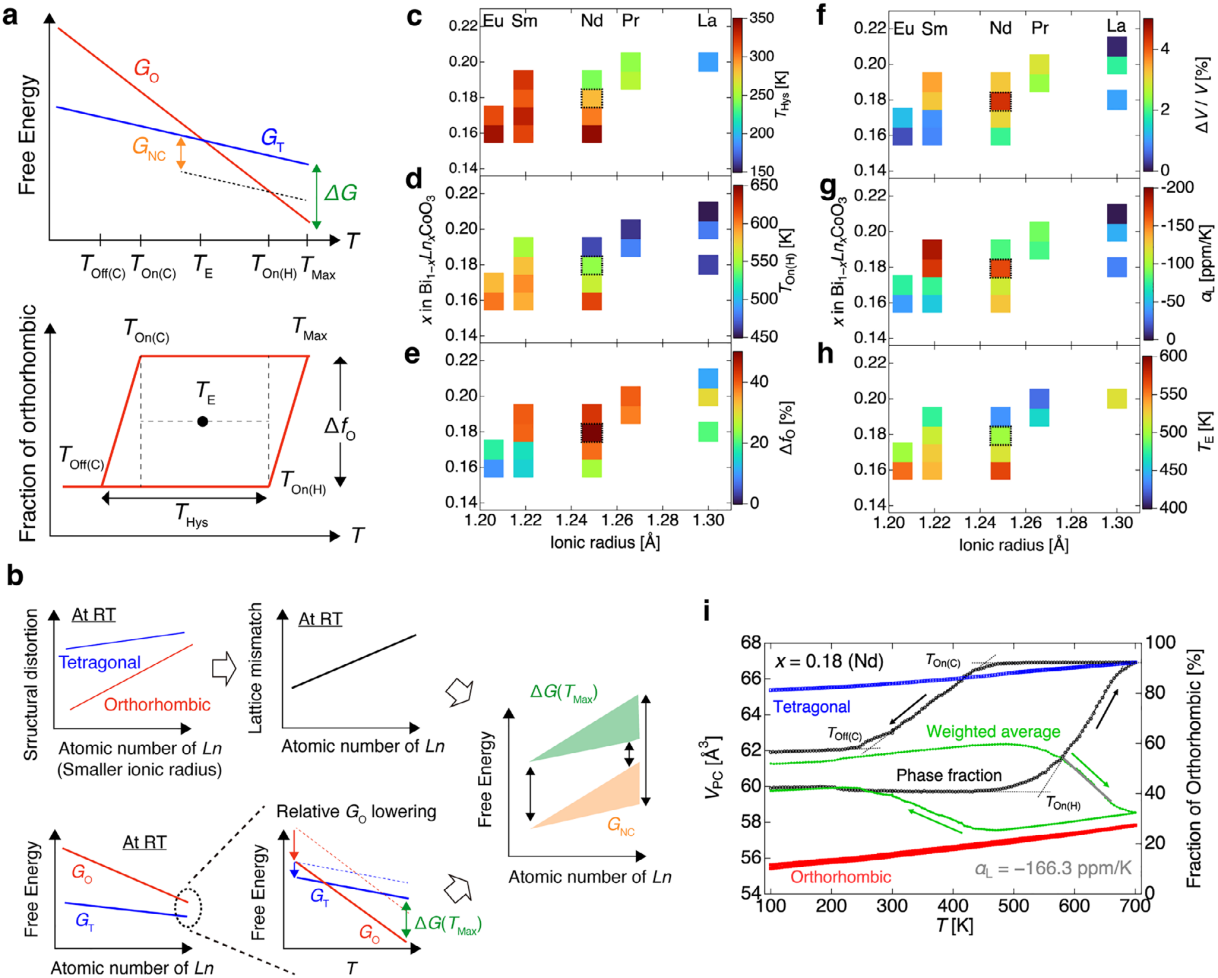
Temperature dependence of weighted average volume in Bi_1−_ *_x_Ln_x_* CoO_3_ indicating the appearance of the largest volume shrinkage in an optimum elemental composition. (a) Schematic image of the relationship between the structural distortions, free energies, and the critical temperatures in martensitic transitions. (b) Schematic image of the expected changes of *G*_NC_ and Δ*G* as a function of the ionic radius of A‐site. The shaded green and orange areas in the figure on the right indicate the ranges where Δ*G* at *T*_Max_ and *G*_NC_ is expected to exist, respectively. Whether their energy differences increase or decrease with decreasing the ionic radius depends on the order of the ionic radius dependence. (c‐h) The width of thermal hysteresis (*T*_Hys_) (c), the onset temperature of the phase transition in the heating process (*T*_On(H)_) (d), the phase‐fraction change (Δ*f*_O_) (e), and the volume shrinkage (Δ*V*/*V*) (f), the liner coefficient of thermal expansion (*α_L_*) (g), the equilibrium temperature (*T*_E_) (h) for Bi_1−_ *_x_Ln_x_* CoO_3_ as functions of ionic radius and concentration of substituting *Ln* ions. The elemental composition surrounded by the black dotted line is the optimum one (Bi_0.82_Nd_0.18_CoO_3_). (i) Temperature dependence of pseudo cubic volumes of tetragonal and orthorhombic phases, fraction of the orthorhombic phase, and weighted average volume for Bi_0.82_Nd_0.18_CoO_3_. *α_L_* = −166.3 ppm/K was obtained by a linear approximation fitting of the weighted average volume in the temperature range depicted as a gray bold line (580–660 K).

Thus, we investigated the temperature variation of the SXRD patterns for *Ln* substituted compounds (*Ln* = Pr, Nd, Sm, and Eu) and their NTE properties. As in the case of La substitution, there are temperature‐induced tetragonal‐to‐orthorhombic phase transitions accompanied by NTE for all of the *Ln*‐substituted compounds near the tetragonal and orthorhombic phase boundaries (Figures S8–S11). Figures 6c‐h respectively plot the width of thermal hysteresis, the onset temperature of the phase transition in the heating process, the phase‐fraction change, the volume shrinkage, the linear coefficient of thermal expansion, and the equilibrium temperature for Bi_1−_ *_x_Ln_x_* CoO_3_ as functions of ionic radius and concentration of substituting *Ln* ions. As the width of thermal hysteresis represents the magnitude of the non‐chemical free energy, the monotonic increase of *T*_Hys_ with decreasing the ionic radius (Figure 6c) is easily acceptable from the ionic radius dependence of *G*_NC_ described above. On the other hand, *T*_On(H)_ is influenced by both Δ*G* and *G*_NC_ contributions (Figure 6a), and the monotonic increase of *T*_On(H)_ with decreasing the ionic radius (Figure 6d) indicates that the ionic radius dependence of *G*_NC_ is larger than that of Δ*G*. Although this result seems to imply that the substitution of smaller ions is disadvantageous for increasing the magnitude of the volume shrinkage, the phase‐fraction change and volume shrinkage exhibit the maximum values at a moderate ionic radius in actual as shown in Figure 6e,f. This is because the absolute value of liner coefficient of thermal expansion exhibits a monotonic increase with decreasing ionic radius (Figure 6g). The nucleation in athermal martensitic transitions often occurs in bursts because a high driving force inhibited by the strain and interfacial energies is released all at once [70, 71]. Therefore, the larger the lattice mismatch between phases is, the more abrupt the transition becomes. The appearance of the minimum *T*_E_ at the moderate ionic radius (Figure 6h) seems to be inconsistent with our explanation considering the relationships between the free energies and the martensitic transition temperatures because the relative energy lowering of the orthorhombic phase must decrease *T*_E_ (Figure 6a). It would be originated from the elastic inner back‐stress occurring on the tip of the domains, which will be explained in detail in the next session. Figure 6i shows the temperature dependence of the lattice parameters, pseudo‐cubic volume, orthorhombic phase fraction, and weighted average volume for Bi_0.82_Nd_0.18_CoO_3_, which showed the largest volume shrinkage. The phase transition was not complete, but the phase‐fraction change of 52%, from 40% to 92%, was much larger than in Bi_1−_ *_x_* La*_x_* CoO_3,_ resulting in a volume shrinkage of 6.1% between 500 and 700 K. This is the largest reported shrinkage in Pb‐free NTE materials.

### 2.5 The Strategy for Improving the Reversibility of NTE

We succeeded in enhancing the volume shrinkage of BiCoO_3_‐based NTE materials by tuning the ionic radius and concentration of substituting *Ln* ions. However, the phase fraction of most Bi_1−_ *_x_Ln_x_* CoO_3_ incompletely returned to the original value after a heat cycle, and the amounts of the residual orthorhombic phase seem to increase with decreasing ionic radius (Figures S10–S12). The phase fraction of Bi_0.8_Pr_0.2_CoO_3_ returned completely in the first heating cycle but incompletely in the second cycle (Figure 7a), implying that the temperature‐induced phase transition is intrinsically reversible and that the irreversible characteristic observed is due to extrinsic effects such as changes of the domain structure and the strain and interfacial free energy. The octahedral distortion parameters, such as the octahedral distortion index *D* [72], the variance of the octahedral angles *σ* ^2^, and the mean octahedral quadratic elongation 〈λ〉 [73] in the tetragonal and orthorhombic phases and the spontaneous polarization in the tetragonal phase are sensitive to the melting of the d*_xy_* orbital ordering. The temperature dependence of these parameters in Bi_0.8_Pr_0.2_CoO_3_ hardly changes in each heat cycle (Figure S13), indicating that the Gibbs free energy itself has not changed after heat cycle. Finally, we discuss the origin of these extrinsic effects and the strategy for improving the reversibility of NTE.

**FIGURE 7:**
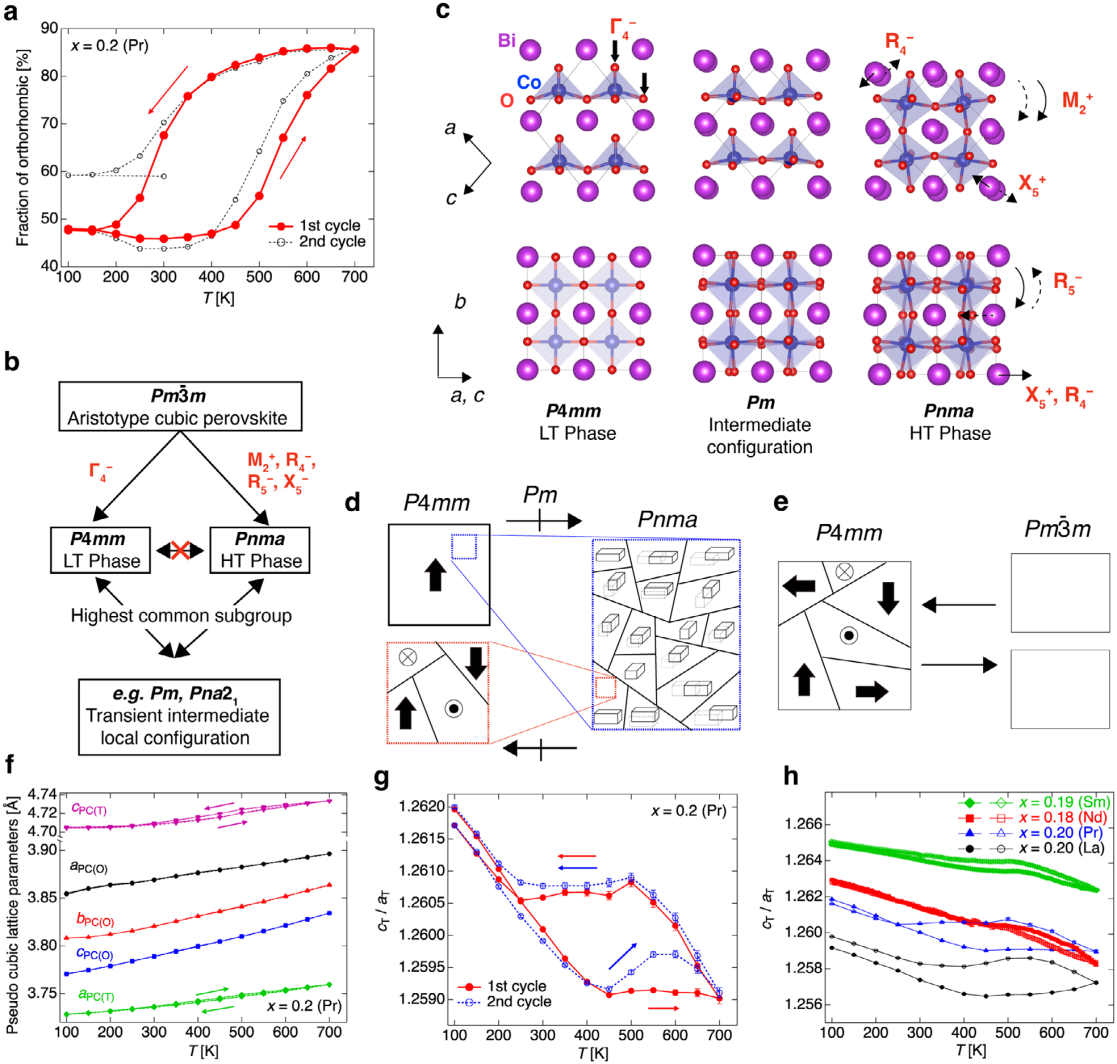
The phase transition without the group‐subgroup relationship decreases the reversibility of the transition. (a) Temperature dependence of pseudo cubic volumes of tetragonal and orthorhombic phases, fraction of the orthorhombic phase, and weighted average volume for Bi_0.8_Pr_0.2_CoO_3_. The phase fraction returned completely in the first heating cycle, implying that the irreversible characteristic is due to extrinsic effects such as changes of the domain structure, strain, and surface free energy. (b) The symmetry relationships between LT tetragonal and HT orthorhombic phases. (c) Transition pathway of the temperature‐induced tetragonal‐to‐orthorhombic phase transition via a polar monoclinic intermediate local configuration with *Pm* space group. Bold linear arrows, solid and dashed curved arrows, solid and dashed linear arrows indicate the polar displacements of O ions, the rotations in front and back octahedra, and the antiparallel displacements in front and back Bi ions, respectively. (d,e) The schematic image of the domain structure change after a heat cycle in the *P*4*mm*‐to‐*Pnma* (d) and *P*4*mm*‐to‐*Pm* $\bar{3}$ *m* (e) phase transitions. The black bold arrow in the figures of the *P*4*mm* phase indicates the direction of the polar axis. In the figure of the *Pnma* phase, the shapes of the rectangular prisms indicate various orientation twins, and the differences from the dotted rectangular prisms indicate antiphase domains. (f) Temperature dependence of pseudo cubic lattice parameters of tetragonal and orthorhombic phases for Bi_0.8_Pr_0.2_CoO_3_. Solid symbols with solid lines and open symbols with dotted lines represent data from the first and second heat cycle, respectively. (g) Temperature dependence of the *c*/*a* ratio for the tetragonal phase of Bi_0.8_Pr_0.2_CoO_3_. (h) Temperature dependence of the *c*/*a* ratio for the tetragonal phases of Bi_0.8_La_0.2_CoO_3_ Bi_0.8_Pr_0.2_CoO_3_, Bi_0.82_Nd_0.18_CoO_3_, and Bi_0.81_Sm_0.19_CoO_3_. Solid symbols with solid lines and open symbols with solid lines represent data from the first heating process and1st cooling process, respectively.

The origin of volume shrinkage in the PbVO_3_‐ and BiCoO_3_‐based NTE materials are essentially the same, the pyramidal‐to‐octahedral coordination change due to the melting of the d*_xy_* orbital ordering. However, the relationships between the symmetries of LT and HT phases in these compounds are significantly different. The temperature‐induced phase transition of the PbVO_3_‐based NTE materials is from a PbTiO_3_‐type polar tetragonal (space group *P*4*mm*) to a nonpolar cubic (space group *Pm* $\bar{3}$ *m*) structures, and there is a group‐subgroup relationship between the symmetries of these phases (Figure 7b). Therefore, although an actual phase transition is of first order due to a large volume difference between the phases, the stabilization of the LT phase can be simply explained by the polar distortion mode described by a $\Gamma_{4}^{-}$ irreducible representation of the *Pm* $\bar{3}$ *m* symmetry. On the other hand, the temperature‐induced phase transition of Bi_1−_ *_x_Ln_x_* CoO_3_ is from the PbTiO_3_‐type polar tetragonal to a GdFeO_3_‐type nonpolar orthorhombic (space group *Pnma*) structure. These structures are not group‐subgroup related, and interestingly, HT phase belongs to a crystal system with lower symmetry. Moreover, these phases have distinct distortion modes: the GdFeO_3_‐type distortion modes, such as in‐phase octahedral tilt modes in [010]_O_ direction ($M_{2}^{+}$), antiphase octahedral tilt modes in [100]_O_ and [001]_O_ directions ($R_{5}^{-}$), and antiparallel A‐site cation displacement modes in [100]_O_ and [001]_O_ directions ($X_{5}^{-}$ and $R_{4}^{-}$), are inactive in the polar tetragonal *P*4*mm* phase (Figure 7c). Although both transitions are of first order, the absence of the group‐subgroup relationship introduces a crucial difference in the domain structure formations in PbVO_3_‐ and BiCoO_3_‐based compounds.

The phase transitions without a group‐subgroup relationship are often seen in the reconstructive phase transitions accompanied by the breaking and rejoining of chemical bonds, such as phase transitions between rocksalt and CsCl structure in NaCl, between rocksalt and zincblende in SiC, and between graphite and diamond in carbon. The temperature‐induced tetragonal‐to‐orthorhombic phase transition of Bi_1−_ *_x_Ln_x_* CoO_3_ also belongs to the reconstructive phase transition. There have been attempts to describe the transition pathways of these transitions with the diffusionless cooperative displacements of the atoms via transient intermediate local configurations, which has the highest common subgroup symmetry of the two end phases and continuously links them, and several transition mechanisms are proposed for these transitions through symmetry analysis, DFT calculations, and MD simulations [74, 75, 76, 77, 78, 79, 80]. We also investigated the transition pathway of the temperature‐induced tetragonal‐to‐orthorhombic phase transition in Bi_1−_ *_x_Ln_x_* CoO_3_ by symmetry analysis and MD simulation. The symmetry analysis using the COMSUBS [76, 77] software proposed only the polar orthorhombic structure with the *Pna*2_1_ space group, which is the same structure of the perovskite‐type CdTiO_3_ and PbNiO_3_, as the candidate of the intermediate configuration. This transition pathway introduces the crystallographic orientation relationship of [001]_T_ // [010]_O_, i.e., the directions of the polar displacement mode ($\Gamma_{4}^{-}$) in the tetragonal phase and the in‐phase octahedral tilt modes ($M_{2}^{+}$) in the orthorhombic phase are parallel. On the other hand, the p2ptrans [78, 79] software proposed another transition pathway via a polar monoclinic structure with the *Pm* space group in addition to the above transition pathway (Table 1). The perpendicular relationship between the in‐phase tilt‐axis and polar‐axis in this transition pathway is consistent with the crystallographic orientation relationship reproduced by MD simulation (Figure 4c and Figure S6). Therefore, although these two transition pathways show almost no difference in the strains and total atomic movements, the latter transition pathway seems to be energetically most plausible. However, both transition pathways may coexist in the real bulk samples because inhomogeneous strain and particle size distributions change the energy barriers of each transition pathway.

**TABLE 1:** Comparison of the candidate transition pathways in the temperature‐induced tetragonal‐to‐orthorhombic phase transition of the BiCoO_3_ derivatives obtained by the p2ptrans software.

Intermediate Configuration	Translation Matrix		Strain Eigenvalues [%]			Total Atomic Displacement [Å / 600 atoms]	Crystallographic Orientation Relationship	Directions of In‐Phase Tilt‐ and Polar‐Axes
	From *P*4*mm*	From *Pnma*	*e*_1_	*e*_2_	*e*_3_			
**Polar** **Orthorhombic** **(S.G. *Pna*2_1_)**	$\left(\begin{array}{ccc} 1 & 1 & 0\\ 1 & -1 & 0\\ 0 & 0 & 2 \end{array}\right)$	$\left(\begin{array}{ccc} 0 & 1 & 0\\ 0 & 0 & 1\\ 1 & 0 & 0 \end{array}\right)$	−18.8	2.6	3.2	686	(1 0 0)_T_ // (‐1 0 1)_O_ [0 1 0]_T_ // [1 0 1]_O_	Parallel
**Polar** **monoclinic** **(S.G. *Pm*)**	$\left(\begin{array}{ccc} 0 & 0 & 2\\ 1 & 1 & 0\\ 1 & -1 & 0 \end{array}\right)$	$\left(\begin{array}{ccc} 1 & 0 & 0\\ 0 & 0 & 1\\ 0 & 1 & 0 \end{array}\right)$	−18.7	2.9	2.9	701	(1 0 0)_T_ // (0 1 0)_O_ [0 1 0]_T_ // [‐1 0 1]_O_	Perpendicular
	$\left(\begin{array}{ccc} 1 & 1 & 0\\ 0 & 0 & 2\\ 1 & -1 & 0 \end{array}\right)$	$\left(\begin{array}{ccc} 0 & 1 & 0\\ 0 & 0 & 1\\ 1 & 0 & 0 \end{array}\right)$	−18.7	2.9	2.9	705	(1 0 0)_T_ // (1 0 1)_O_ [0 1 0]_T_ // [0 1 0]_O_	

The transformation to the structure with the common subgroup symmetry introduce the symmetry lowering in the processes of both forward and reverse phase transitions. Therefore, heat cycles in these transitions increase the number of domains in a particle. For example, the most plausible transition pathway in BiCoO_3_ derivatives via the polar monoclinic intermediate local configuration with *Pm* space group proposed above induces 16 domains (4 antiphase domains per 4 orientation twins) upon heating and 4 domains (4 orientation twins) upon cooling, resulting in the possibility of the splitting into up to 64 domains in a single heat cycle (Figure 7d). On the other hand, in the case of the temperature‐induced tetragonal‐to‐cubic phase transition in PbVO_3_ derivatives, the transformation twins generated by the transition upon cooling disappear by re‐heating to HT cubic phase (Figure 7e). The increase of the number of domain structures in particles increase the possibility of irreversible plastic deformations, resulting in the lowering of the reversibility in the transitions [81, 82, 83]. High driving force in the burst‐type nucleation increases the possibility of the appearance of various domains and transition pathways, which is consistent with the lowering of the reversibility with decreasing the ionic radius of substituting *Ln* ions (Figure S12).

These results may seem to imply that the present NTE compound is not suitable for practical applications. However, transitions without a group‐subgroup relationship between the phases also show some degrees of domain structural reversibility. The Fe‐Mn‐Si shape memory alloys utilizing the strain‐induced martensitic transition between *γ*‐austenite with a fcc structure and *ε*‐martensite with a hcp structure have been commercialized due to their significant reversibility of domain structure [84]. Such reversibility is thought to be due to the elastic inner back stress accommodated on the growing tip of the martensite plates, which assists the crystallographic path in the reverse transition the same as those in the forward transition [85, 86, 87, 88]. As this elastic back‐stress promotes the reverse transition, *T*_On(H)_ is lower than *T*_On(C)_ in some thermoelastic martensitic transitions [89]. Interestingly, the same phenomenon is observed in Bi_0.8_La_0.2_CoO_3_ (Figure 3b and Figure S4), indicating that elastic stress is one of the key driving forces of the temperature‐induced tetragonal‐to‐orthorhombic phase transition in BiCoO_3_ derivatives. The phase reversibility is also explained well by this elastic stress.

In the martensitic transitions, the inner stresses associated with the transformation causes the deviation of the lattice parameters of martensite and austenite in the phase coexistence region from the values extrapolated from the thermal expansion coefficients, and the magnitude of this deviation also varies with a heat cycle [90]. Similar anomalies in the lattice parameters are also observed in Bi_0.8_Pr_0.2_CoO_3_. Figure 7f shows the temperature dependence of the pseudo‐cubic lattice parameters for the tetragonal and orthorhombic phases of Bi_0.8_Pr_0.2_CoO_3_ in the first and second heat cycles. The lattice parameters in the tetragonal phase were found to exhibit non‐monotonic changes that differ slightly in each cycle. The anomalies of the lattice parameter in the tetragonal phase can be systematically explained from the temperature dependence of the *c*/*a* ratio for the tetragonal phase (Figure 7g). The *c*/*a* ratio decreased with increasing temperature in the region where the phase fraction didn't change, which is consistent with the destabilization of the polar tetragonal phase upon heating. However, the *c*/*a* ratio showed plateau behavior when the phase fraction of the orthorhombic phase started to increase, and similar behavior was also observed in the first cooling process. Interestingly, this plateau behavior in the *c*/*a* ratio transformed into bump behavior in the second heating process. In the phase coexistence region, there are various components affecting the lattice parameter, such as the phase strain introduced by the transition, crystal defects, and thermal misfit strain. Therefore, it is difficult to separate each contribution. However, considering the decrease of the phase reversibility after the second heating cycle, these plateau and bump behaviors would be roughly originated from the accommodation of the elastic stress and the release of the elastic stress due to the plastic deformation (plastic relaxation) in the untransformed LT tetragonal phase, respectively. Figure 7h shows the temperature dependence of the *c*/*a* ratio for Bi_0.8_La_0.2_CoO_3_, Bi_0.82_Nd_0.18_CoO_3_, and Bi_0.81_Sm_0.19_CoO_3_. The *c*/*a* ratio of Bi_0.8_La_0.2_CoO_3_ also exhibited large thermal hysteresis with a wide plateau region, while those of Bi_0.82_Nd_0.18_CoO_3_ and Bi_0.81_Sm_0.19_CoO_3_ continuously decreased with increasing temperature after showing a narrow plateau region. The narrowing of the plateau region indicates that the plastic relaxation is more dominant than the accommodation of the elastic stress, which is consistent with the large lattice mismatch in the compounds with a small *Ln* ion. The incomplete phase reversibility of Bi_0.8_Pr_0.2_CoO_3_ in the second heat cycle would be due to the decrease of the ability to accommodate elastic stress resulting from the miniaturization of the domain structures. The elastic inner back‐stress promotes the reverse transition, resulting in the increase of *T*_On(C)_. As the plastic relaxation releases the elastic stress, the accumulated elastic stress decreases with decreasing ionic radius of the substituting *Ln* ions. On the other hand, *T*_On(H)_ increases with decreasing ionic radius. As *T*_E_ is defined as (*T*_On(H)_ + *T*_On(C)_)/2, *T*_E_ is lowest at an intermediate ionic radius. Due to the presence of large elastic inner stress, BiCoO_3_‐based NTE materials are also expected to achieve practical application by reducing the amount of irreversible plastic deformation through reduction of lattice mismatch.

For achieving the complete temperature‐induced phase transition, the increase of the relative Gibbs free energy and small lattice mismatch between the tetragonal and orthorhombic phases are important. The increase in the GdFeO_3_‐type distortions is advantageous for the phase‐fraction change but disadvantageous for the temperature hysteresis and phase reversibility, while the increase in the polar distortion is always disadvantageous. Therefore, the reduction of the *c*/*a* ratio is undoubtedly required, and the resulting increase of the Gibbs free energy of the tetragonal phase reduces the need for an increase in the GdFeO_3_‐type distortion. Even a small reduction in the *c*/*a* ratio may alter the formation process of domain structures, resulting in the drastic improvement of NTE. The comprehensive studies, including metadynamics simulations, large‐scale MD simulations with ab initio accuracy, and the observation of domain structures are required to clarify the details of such complicated transition mechanisms accompanied by domain structural changes, which is our future challenge of experimental and theoretical research.

## 3 Conclusion

The largest volume shrinkage in Pb‐free NTE materials was achieved in perovskite‐type BiCoO_3_ derivatives. BiCoO_3_ has a giant tetragonal distortion, CoO_5_ pyramidal coordination, and a large pseudo‐cubic volume due to the cooperative effect of the stereochemical activity of the Bi^3+^ lone‐pair electrons and the d*_xy_* orbital ordering in HS Co^3+^ with a d^6^ electronic configuration. The substitution of the lone‐pair inactive *Ln* ^3+^ ions for Bi^3+^ decreases the stereochemical activity, which leads to melting of the d*_xy_* orbital ordering upon heating, accompanied by a tetragonal‐to‐orthorhombic structural transition and coordination change from the CoO_5_ pyramid with HS Co^3+^ to the CoO_6_ octahedron with the LaCoO_3_‐type Co^3+^ spin state (IS or the HS‐LS mixed spin state). This temperature‐induced phase transition occurs in various *Ln*‐substituted compounds near the tetragonal, and orthorhombic phase boundary, resulting in a large volumetric NTE due to the pyramidal‐to‐octahedral coordination changes. Bi_0.82_Nd_0.18_CoO_3_ shows the largest volume shrinkage, 6.1%, which is more than twice that of commercial NTE materials, although the fraction of the orthorhombic phase changes only by 52%. As the potential volume shrinkage of BiCoO_3_‐based NTE materials is 14%, which was obtained from the volume difference of tetragonal and orthorhombic phases at 600 K in Bi_0.8_La_0.2_CoO_3_, a further improvement in shrinkage performance can be expected by enhancing the magnitude of the phase‐fraction change. Although the transformation between the tetragonal and orthorhombic phases are incredibly complicated due to the absence of the group‐subgroup relationship between the LT and HT phases, the reduction in a *c*/*a* ratio would be effective in improving the NTE properties. By analogy from studies on the PbVO_3_‐based NTE materials, the *c*/*a* ratio smaller than 1.2 seems to be required to achieve a complete temperature‐induced phase transition [53]. Efforts to decrease the *c*/*a* ratio further are underway. The nucleation process in the reconstructive phase transition, where no group‐subgroup relationship exists between the initial and final phases, is far more complicated than that in the transition with the group‐subgroup relationship, making it difficult to tune the properties of NTE induced by the reconstructive phase transition. However, the development of the reconstructive‐type NTE materials is essential to further increase the magnitude of the volume contraction because significantly large volume changes usually involve the breaking and reformation of chemical bonds. Therefore, the present work, the achievement of the reversible NTE in the reconstructive phase transition, is an important stepping stone to the development of new colossal NTE materials.

## 4 Experimental Section

Polycrystalline samples of Bi_1−_ *_x_Ln_x_* CoO_3_ (*Ln* = La, Pr, Nd, Sm, and Eu) were synthesized by mixing Bi_2_O_3_, Co_3_O_4_, La_2_O_3_, Pr_6_O_11_, Nd_2_O_3_, Sm_2_O_3_, and Eu_2_O_3_, packing them in a platinum capsule, and treating them at 8 GPa and 1473 K for 30 min in a cubic‐anvil high‐pressure (HP) apparatus. KClO_4_ was put at the bottom and top of each capsule as an oxidizer. The obtained samples were washed with distilled water to remove the residual KCl.

The SXRD data were collected with large Debye–Scherrer cameras installed on the BL02B2 [91, 92] and BL13XU beamlines of SPring‐8. The diffraction data were analyzed with the Rietveld method using the TOPAS software [93].

The XAS measurements around the Co L‐edges were performed at the soft X‐ray beamline BL27SU of SPring‐8. The XAS data were collected at 300 K by using the TEY and PFY methods, and the IPFY data were obtained from the inverse of the oxygen PFY spectra in the Co L‐edges region. The powder samples were uniformly embedded on indium films or carbon tapes.

The temperature dependence of the magnetic susceptibility of Bi_0.8_La_0.2_CoO_3_ was measured with a SQUID magnetometer (Quantum Design, MPMS 5) in an external magnetic field of 1 kOe.

The thermo‐mechanical properties of Bi_0.82_La_0.18_CoO_3_ were investigated using MD simulations based on the CHGNet machine learning force field. The simulations were performed using the Atomic Simulation Environment (ASE) framework [94], starting from an initial crystal structure with 4 × 4 × 4 pseudocubic unit cells. To study the material's thermal expansion, simulations were conducted within the isothermal‐isobaric (NPT) ensemble. The system was incrementally heated from 1 to 1000 K in steps of 1 K. At each temperature, the system was allowed to equilibrate for 0.1 ps, employing a time step of 1.0 fs. The external pressure was maintained at 1.0 bar. Standard thermostat and barostat algorithms within ASE were used for temperature and pressure control, allowing for anisotropic fluctuations of the simulation cell. In addition to temperature studies, the effect of hydrostatic pressure for BiCoO_3_ was investigated at a fixed low temperature of 10 K. Using a similar NPT ensemble setup, the external pressure was incrementally increased from 0.0 to 5.0 GPa in steps of 0.1 GPa. At each applied pressure, the system was equilibrated for 1 ps with a 1.0 fs time step, employing the same thermostat and barostat settings as in the temperature‐dependent simulations. Throughout all simulations, system properties including potential energy, instantaneous temperature, cell volume, and lattice parameters were continuously monitored and recorded.

The transition pathway of the temperature‐induced tetragonal‐to‐orthorhombic phase transition in Bi_1−_ *_x_Ln_x_* CoO_3_ were investigated by using COMSUBS [76, 77] and p2ptrans [78, 79] software. These two types of software find the plausible transition pathway of the reconstructive phase transition with different algorithms. The former software first finds the supercell with common subgroups of initial and final structures and then evaluates the strains and atomic movements in the transition by using the resulting supercell. On the other hand, the latter software first builds the atom‐to‐atom map between initial and final structures that minimizes the displacement of all the atoms in a large‐scale supercell, 400 unit cells of tetragonal phases in the present analysis, and retrieves the periodicity from the resulting atom‐to‐atom map. As there are an infinite number of structures with a common subgroup of initial and final structures, the candidate structures obtained by these types of software are slightly different. The crystallographic parameters of BiCoO_3_ and Bi_0.6_La_0.4_CoO_3_ refined from the Rietveld analysis (Table S1) were used as those of the initial (tetragonal) and final (orthorhombic) phases, respectively.

[CCDC 2440749, 2440750, and 2440751 contain the supplementary crystallographic data for this paper. These data can be obtained free of charge from The Cambridge Crystallographic Data Centre via www.ccdc.cam.ac.uk/data_request/cif.]

## Author Contributions

**Kazuki Takahashi**: formal analysis, investigation, validation, visualization, data curation. **Takafumi Yamamoto**: investigation. **Kano Hatayama**: investigation. **Shogo Wakazaki**: formal analysis, software, methodology, validation, visualization. **Yuki Sakai**: conceptualization, formal analysis, funding acquisition, methodology, investigation, project administration, software, visualization, validation, writing – original draft, data curation. **Takumi Nishikubo**: funding acquisition, investigation. **Teppei Nagase**: investigation. **Masaichiro Mizumaki**: investigation, supervision, methodology. **Jun Miyake**: investigation, software. **Takehiro Koike**: investigation, software. **Masaki Azuma**: conceptualization, resources, funding acquisition, project administration, supervision, methodology, validation, writing – review and editing, visualization.

## Funding

This work was partially supported by JSPS KAKENHI (grant nos. JP19H05625, JP24K08036, JP24K17509, and JP24H00374), JST CREST (Grant Number. JPMJCR22O1), Grant for Basic Research Projects from the Sumitomo Foundation (Grant Number. 190798), the Kanagawa Institute of Industrial Science and Technology, Design and Engineering by Joint Inverse Innovation for Materials Architecture of the Ministry of Education, Culture, Sports, Science and Technology, and the Collaborative Research Project of Materials and Structures Laboratory, Institute of Integrated Research, Institute of Science Tokyo.

## Conflicts of Interest

The authors declare no conflicts of interest.

## Supporting information




**Supporting File 1**: advs77022‐sup‐0001‐SuppMat.pdf.

**Supporting File 2**: advs77022‐sup‐0002‐VideoS1‐S2.zip.

**Supporting File 3**: advs77022‐sup‐0003‐DataFiles.zip.

## Data Availability

The data that support the findings of this study are available from the corresponding author upon reasonable request.
