# Supplementary material for: 6.1% Volumetric Negative Thermal Expansion Induced by the Reversible Reconstructive Phase Transition in Pb‐Free Bi1− xLnx CoO3 (Ln: Lanthanoid)
Source: Adv Sci (Weinh). 2026 Sep 3;13(59):e77022. doi: 10.1002/advs.77022 (PMC13541412; doi:10.1002/advs.77022)
Supplement: Supplementary file 1 [file ADVS-13-e77022-s001.pdf]

Supporting Information

6.1% Volumetric Negative Thermal Expansion Induced by the Reversible Reconstructive Phase Transition in Pb-Free $Bi_{1-x}Ln_xCoO_3$ (*Ln*: Lanthanoid)

Yuki Sakai, Kazuki Takahashi, Shogo Wakazaki, Takumi Nishikubo, Jun Miyake, Takehiro Koike, Teppei Nagase, Takafumi Yamamoto, Kano Hatayama, Masaichiro Mizumaki and Masaki Azuma**

Dr Y. Sakai, Dr T. Nishikubo, Prof M. Azuma
Kanagawa Institute of Industrial Science and Technology
705-1 Shimoimaizumi, Ebina, Kanagawa 243-0435, Japan
E-mail: y_sakai@cross.or.jp

Dr Y. Sakai, K. Takahashi, S. Wakazaki, Dr T. Nishikubo, J. Miyake, T. Koike, T. Nagase, Prof T. Yamamoto, K. Hatayama, Prof M. Azuma
Materials and Structures Laboratory
Institute of Integrated Research,
Institute of Science Tokyo
4259 Nagatsuta-cho, Midori-ku, Yokohama, Kanagawa 226-8501, Japan
E-mail: mazuma@msl.iir.isct.ac.jp

Dr T. Nishikubo, Prof M. Azuma
Research Center for Autonomous Systems Materialogy (ASMat)
Institute of Integrated Research
Institute of Science Tokyo
4259 Nagatsuta-cho, Midori-ku, Yokohama, Kanagawa 226-8501, Japan

Prof M. Mizumaki
Japan Synchrotron Radiation Research Institute (JASRI)
1-1-1 Kouto, Sayo-cho, Sayo-gun, Hyogo 679-5198, Japan

Prof M. Mizumaki
Faculty of Advanced Science and Technology
Kumamoto University
2-39-1 Kurokami Chuo-ku, Kumamoto, Kumamoto 860-8555, Japan

Table S1. Crystallographic parameters of $BiCoO_3$ and $Bi_{0.6}La_{0.4}CoO_3$ at 300 K.

atoms	site	*g*	*x*	*y*	*z*	$100\times U_{iso}$ [Å^2]
**$BiCoO_3$[a]**						
Tetragonal phase						
Bi	1a	1	0	0	0	0.54(1)
Co	1b	1	0.5	0.5	0.5734(4)	0.40(5)
O1	1b	1	0.5	0.5	0.2167(15)	0.20(14)
O2	2c	1	0.5	0	0.7211(11)	0.20(14)
**$Bi_{0.6}La_{0.4}CoO_3$[b]**						
Tetragonal phase						
Bi	1a	0.6	0	0	0	0.87(13)
La		0.4				
Co	1b	1	0.5	0.5	0.5776(29)	0.63(fix)
O1	1b	1	0.5	0.5	0.2565(127)	1.27(fix)
O2	2c	1	0.5	0	0.7015(83)	1.27(fix)
Orthorhombic phase						
Bi	4c	0.6	0.0383(2)	0.25	0.9932(8)	1.34(3)
La		0.4				
Co	4b	1	0	0	0.5	0.33(6)
O1	4c	1	0.4840(35)	0.25	0.0849(54)	1.27(fix)
O2	8d	1	0.2883(48)	0.0345(36)	0.7206(50)	1.27(fix)
Rhombohedral phase						
Bi	4c	0.6	0	0	0.25	0.63(fix)
La		0.4				
Co	4b	1	0	0	0	0.63(fix)
O1	8d	1	0.5243(235)	0	0.25	1.27(fix)

[a] Space group *P*4*mm* (99), Z =1, a = 3.72899(2) Å, c = 4.72194(5) Å for tetragonal phase; R_{wp} = 7.02%, R_p = 4.89%, R_B = 1.81%, S = 2.69.

[b] Space group *P*4*mm* (99), Z =1; a = 3.74391(20) Å, c = 4.71123(44) Å, 15.3(2)wt% for tetragonal phase; Space group *Pnma* (62), Z = 4; a = 5.44433(18) Å, b = 7.67244(17) Å, c = 5.40858(18) Å, 73.1(3)wt% for orthorhombic phase; Space group $R\bar{3}c$ (167), Z = 6; a = 5.44538(56) Å, c = 13.11252(172) Å, 11.7(2)wt% for rhombohedral phase; R_{wp} = 6.26%, Rp = 4.94%, R_B = 1.36% (tetragonal phase), 1.27% (orthorhombic phase), and 1.54% (rhombohedral phase), S = 0.98. Occupation factors of all sites were fixed.

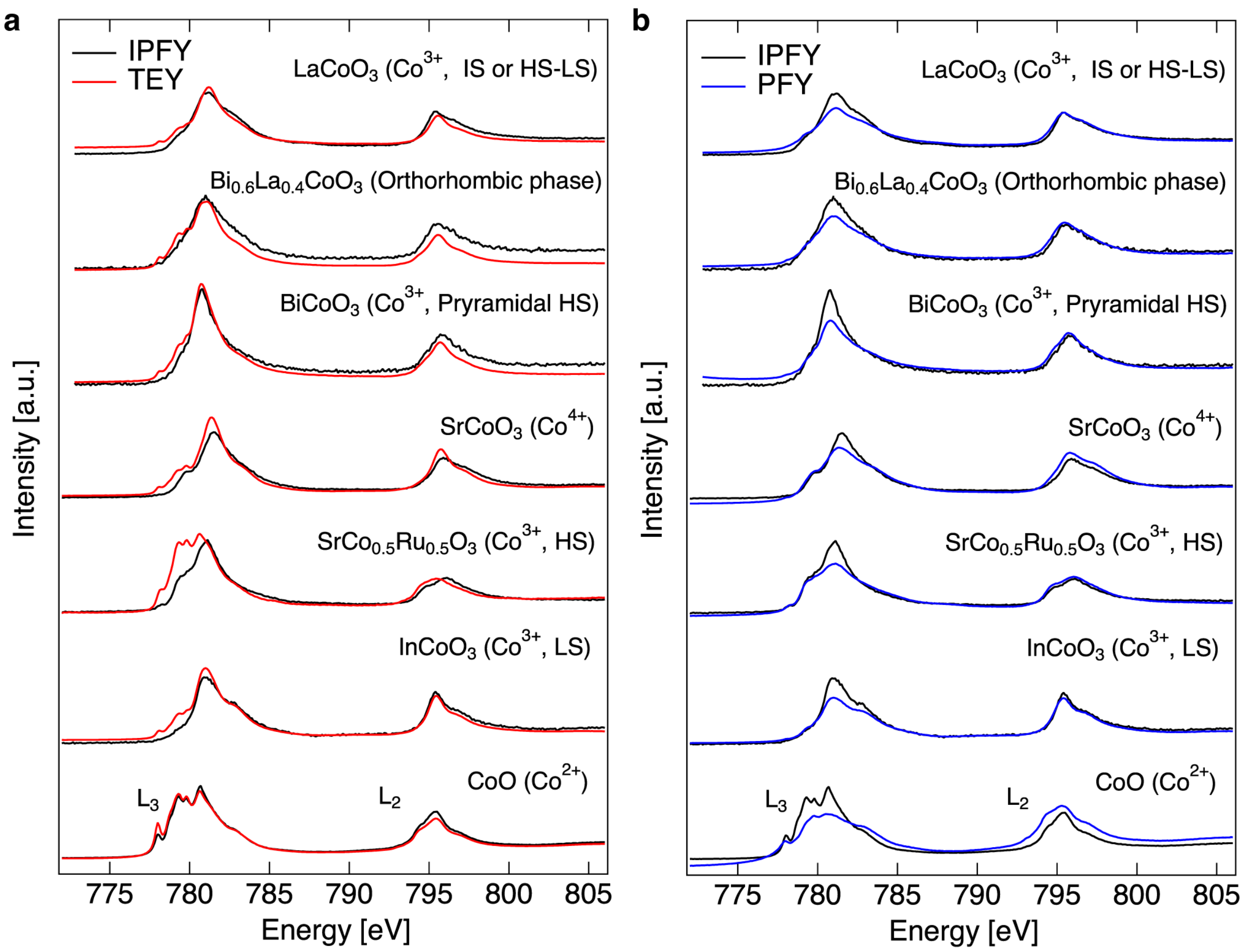

Figure S1. Comparisons of Co L-edge XAS spectra of $Bi_{0.6}La_{0.3}CoO_3$ and some Co standard materials at RT between IPFY, TEY (a), and PFY (b) methods. IPFY spectra enable to avoid contaminations of Co^{2+} components from the surfaces and distortions due to the self-absorption effects.

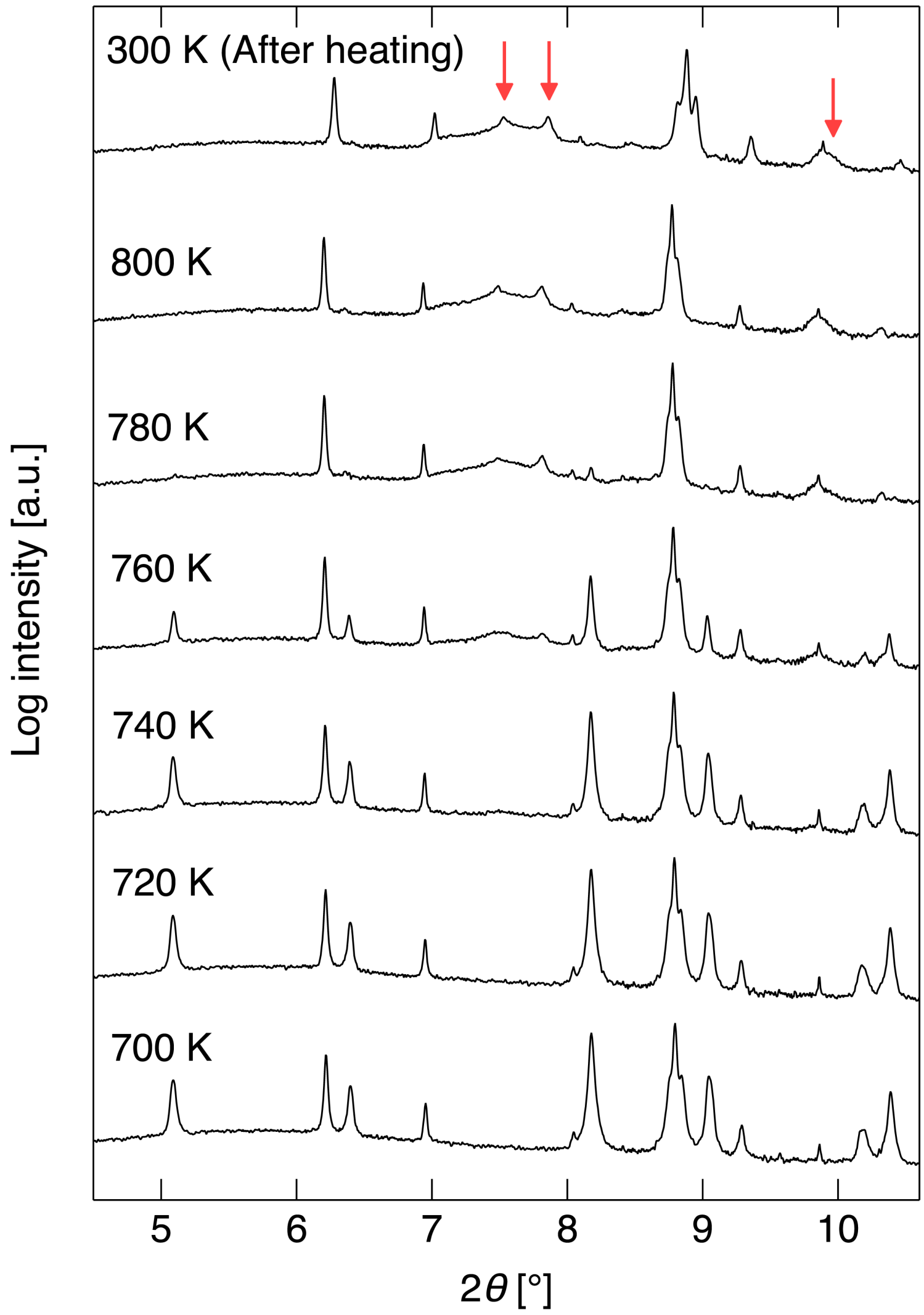

Figure S2. The SXRD patterns of $Bi_{0.8}La_{0.2}CoO_3$ at various temperatures upon heating above 700 K. The wavelengths used are 0.420264 Å. The red arrows indicate the Bragg peaks from decomposition compounds.

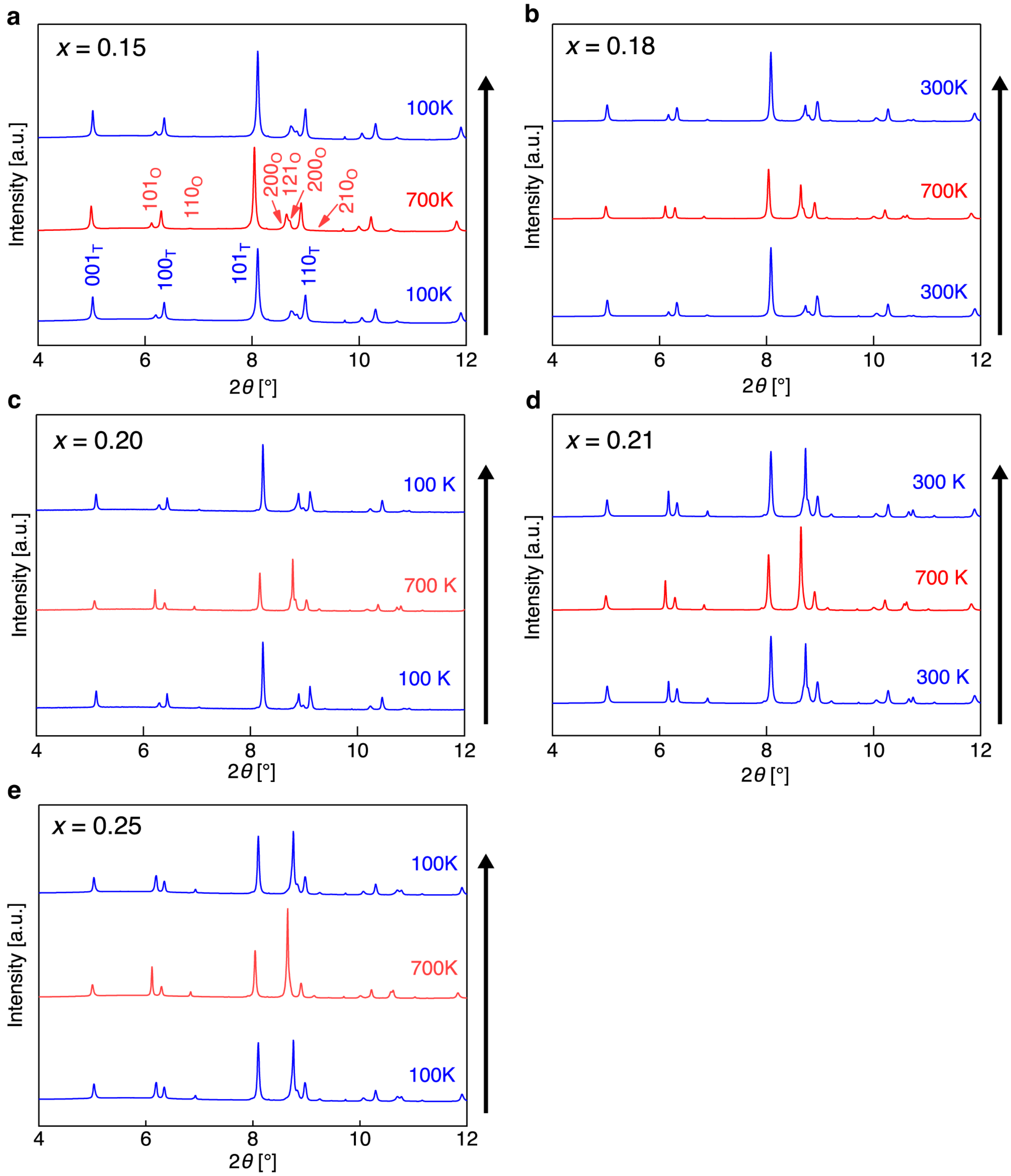

Figure S3. The SXRD patterns of $Bi_{1-x}La_xCoO_3$ at minimum and maximum temperatures upon heating and cooling. The wavelengths used are 0.413064 (b, d), 0.413040 (a, e), and 0.420264 (c) Å.

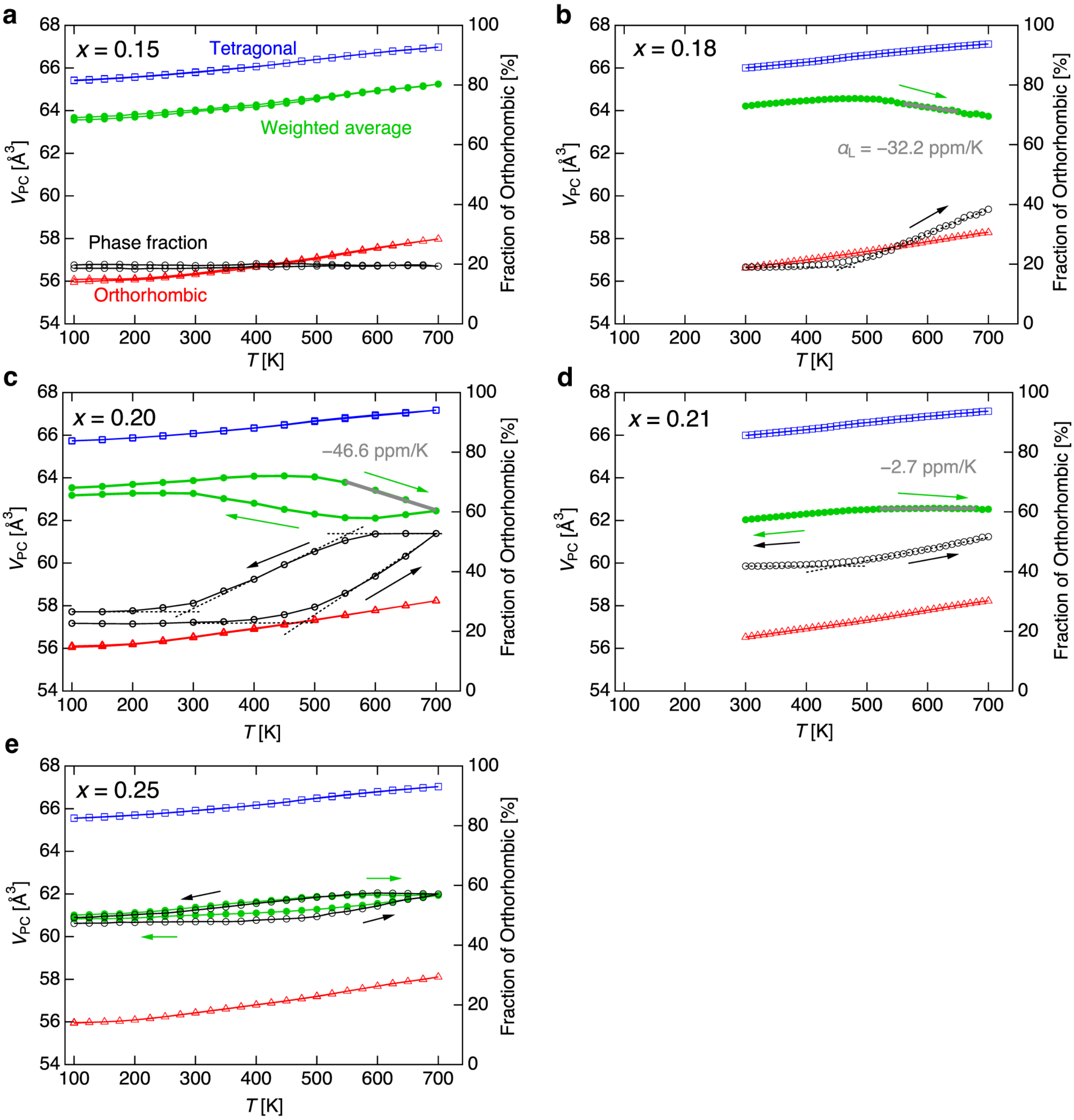

Figure S4. The temperature dependence of pseudo cubic volumes of the tetragonal and orthorhombic phases, the fraction of the orthorhombic phase, and weighted average volume for $Bi_{1-x}La_xCoO_3$. Gray bold lines on the weighted average volume indicate results of linear approximation fittings used to calculate the α_Ls.

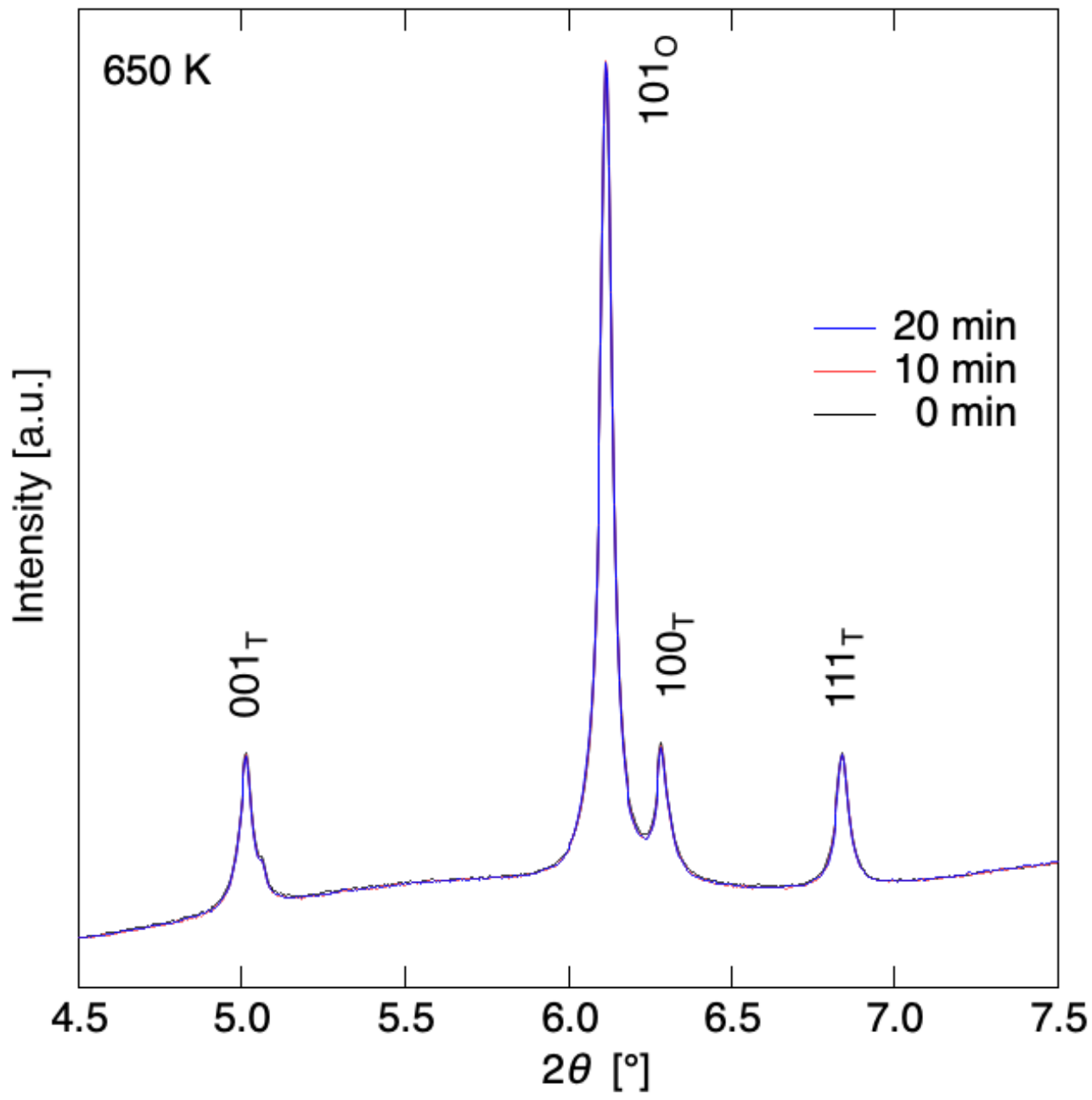

Figure S5. The time-lapse SXRD patterns of $Bi_{0.8}La_{0.2}CoO_3$ at 650 K. The wavelengths used are 0.413040 Å.

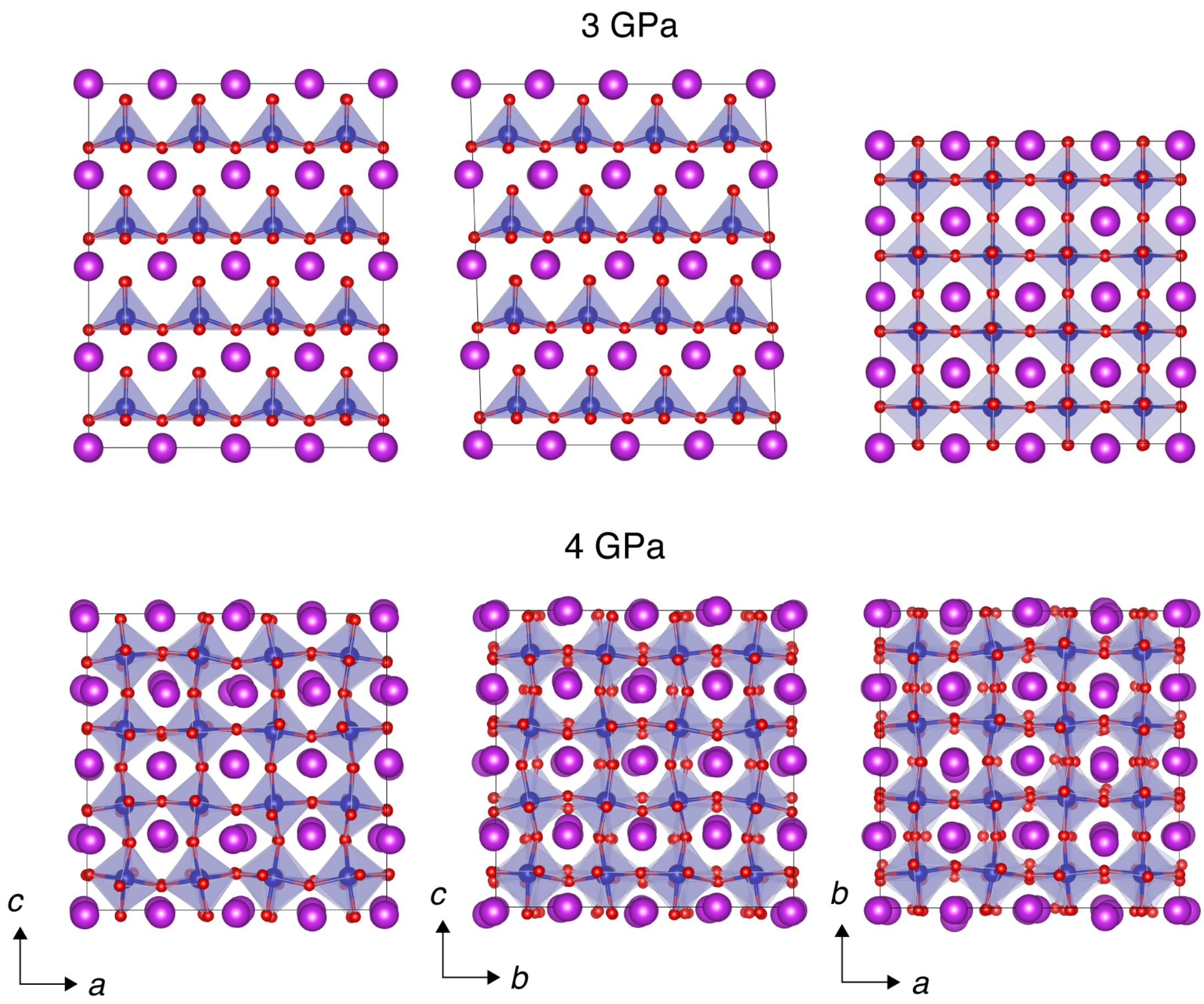

Figure S6. Crystal structures of $BiCoO_3$ reproduced by MD simulations at 3 and 4 GPa. Purple, blue, and red spheres represent Bi, Co, and O atoms, respectively.

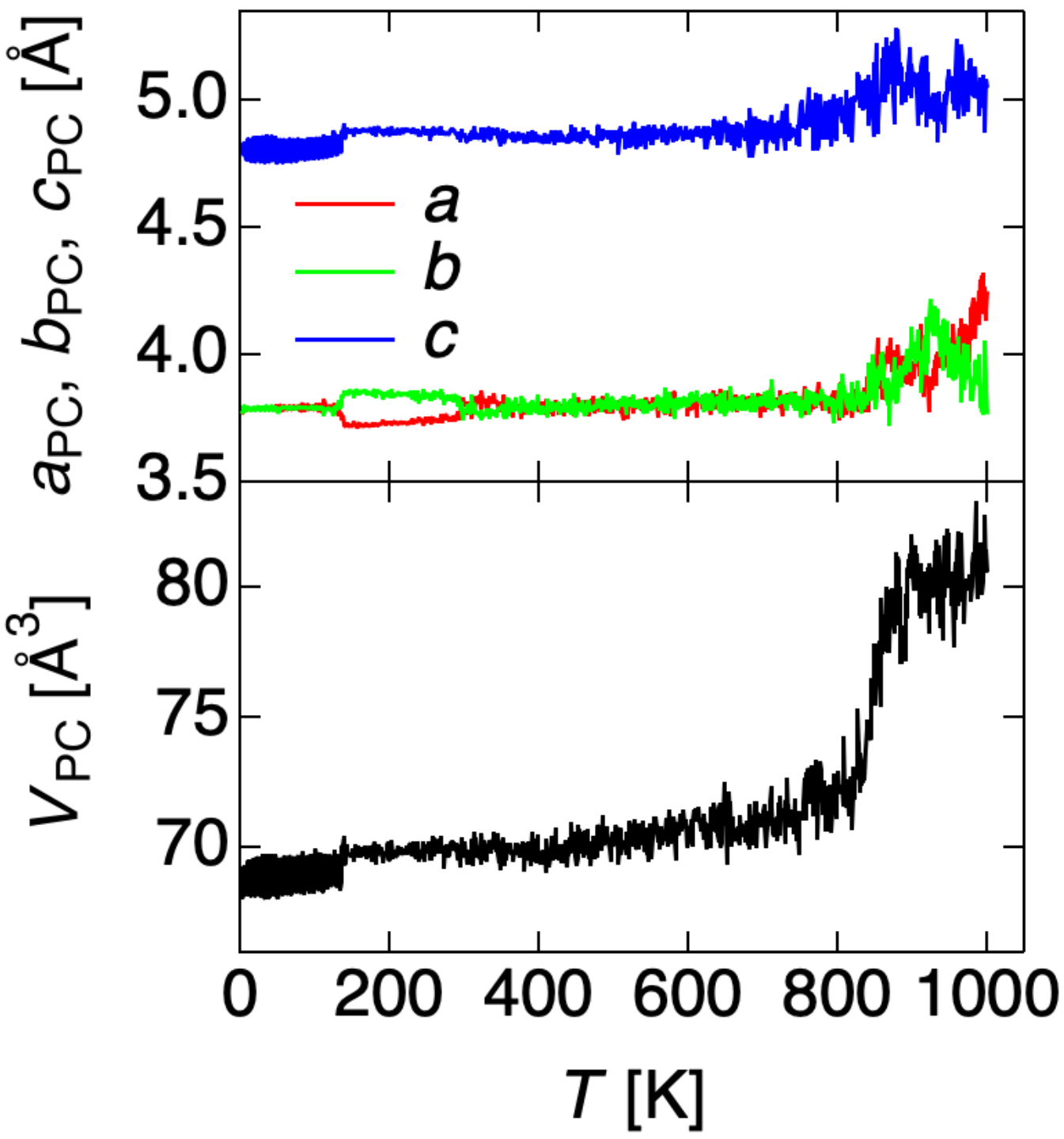

Figure S7. Simulated temperature dependence of the pseudo cubic lattice parameters and pseudo-cubic unit cell volume for $BiCoO_3$. The large volume expansion above 800 K, indicates the decompositions of perovskite-type structures.

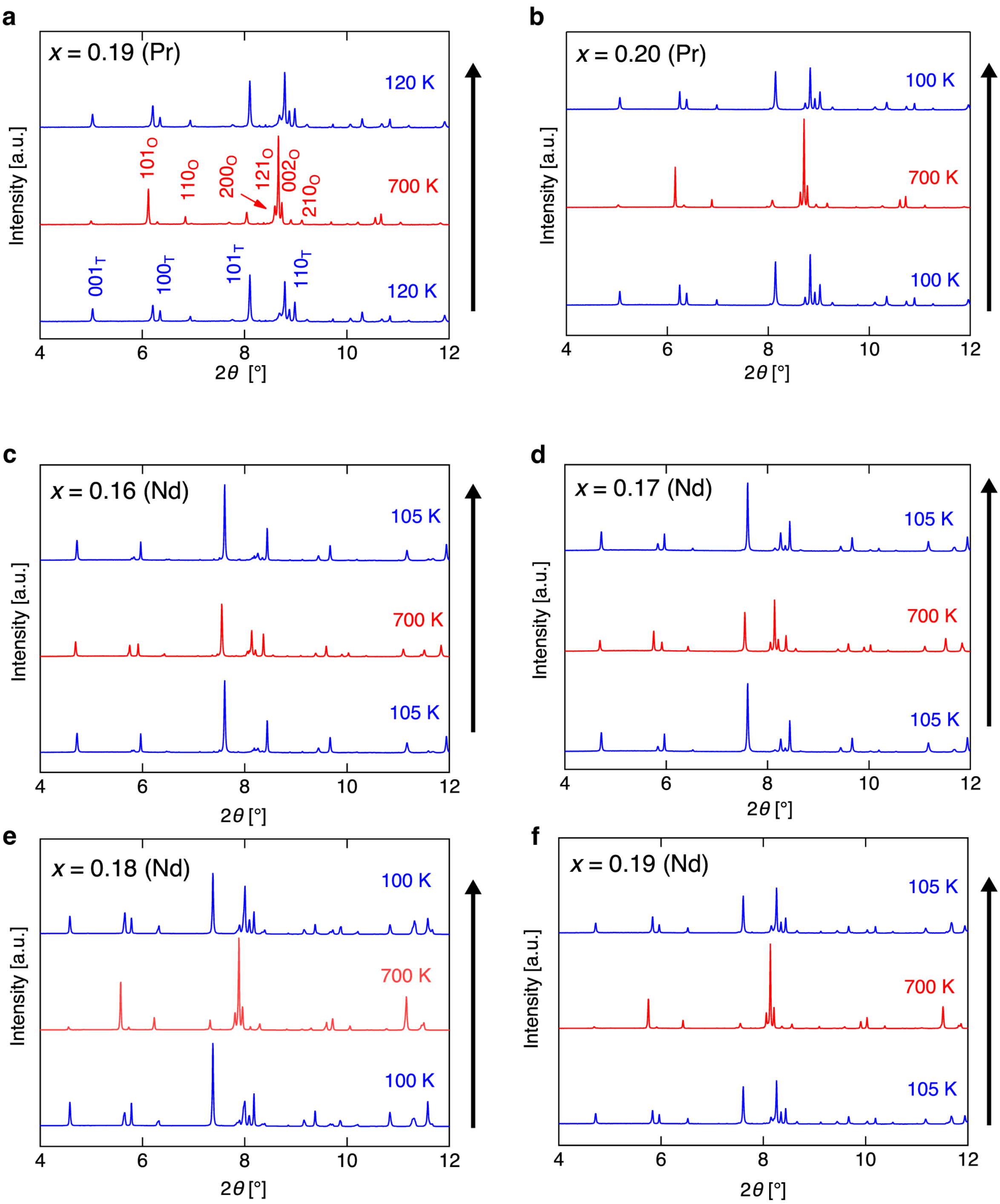

Figure S8. The SXRD patterns of $Bi_{1-x}Pr_xCoO_3$ (a-b) and $Bi_{1-x}Nd_xCoO_3$ (c-f) at minimum and maximum temperatures upon heating and cooling. The wavelengths used are 0.4144167 (b), 0.354362 (e), and 0.387621 (a, c, d, f) Å.

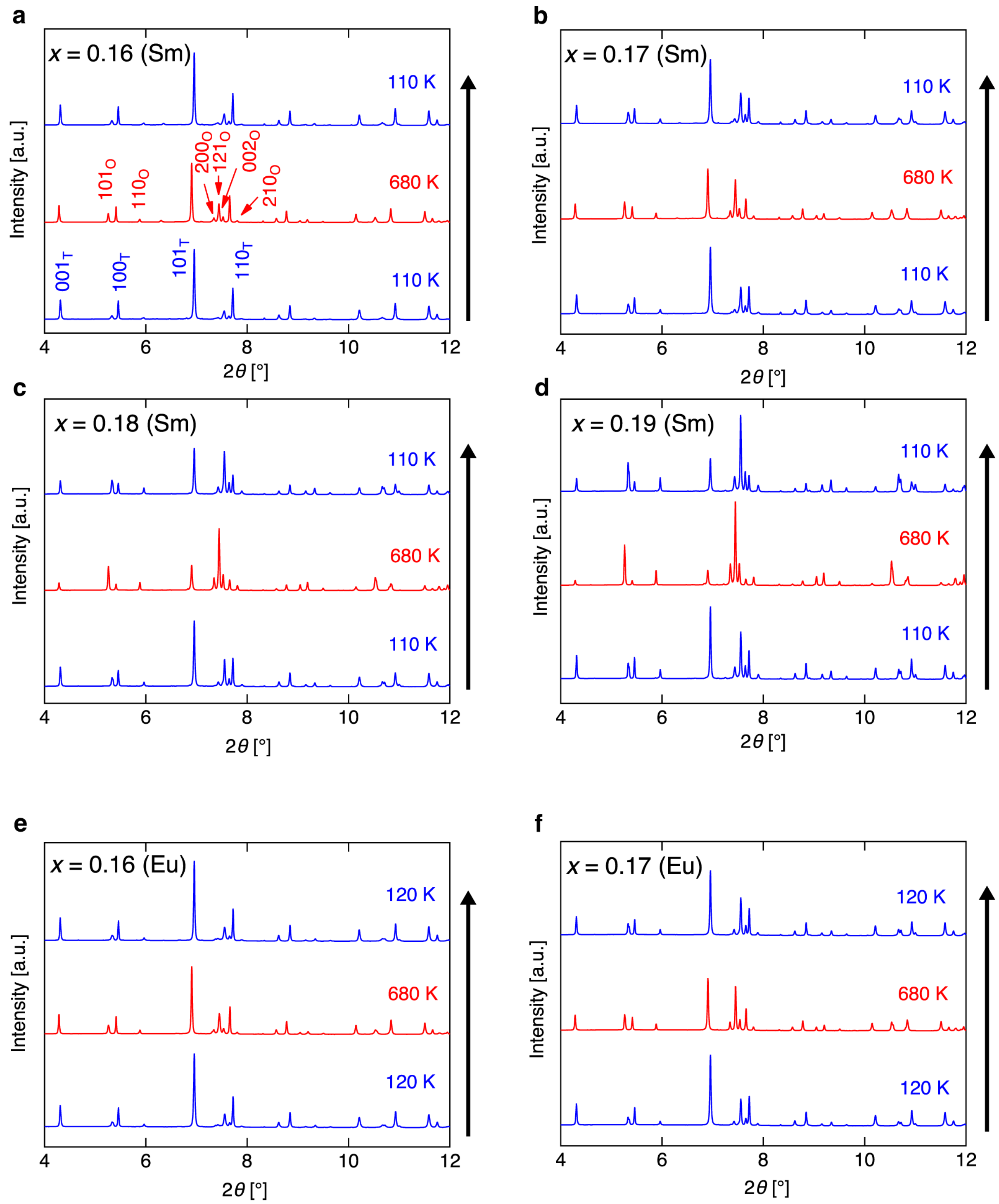

Figure S9. The SXRD patterns of $Bi_{1-x}Sm_xCoO_3$ (a-d) and $Bi_{1-x}Eu_xCoO_3$ (e-f) at minimum and maximum temperatures upon heating and cooling. The wavelength used is 0.354362 Å.

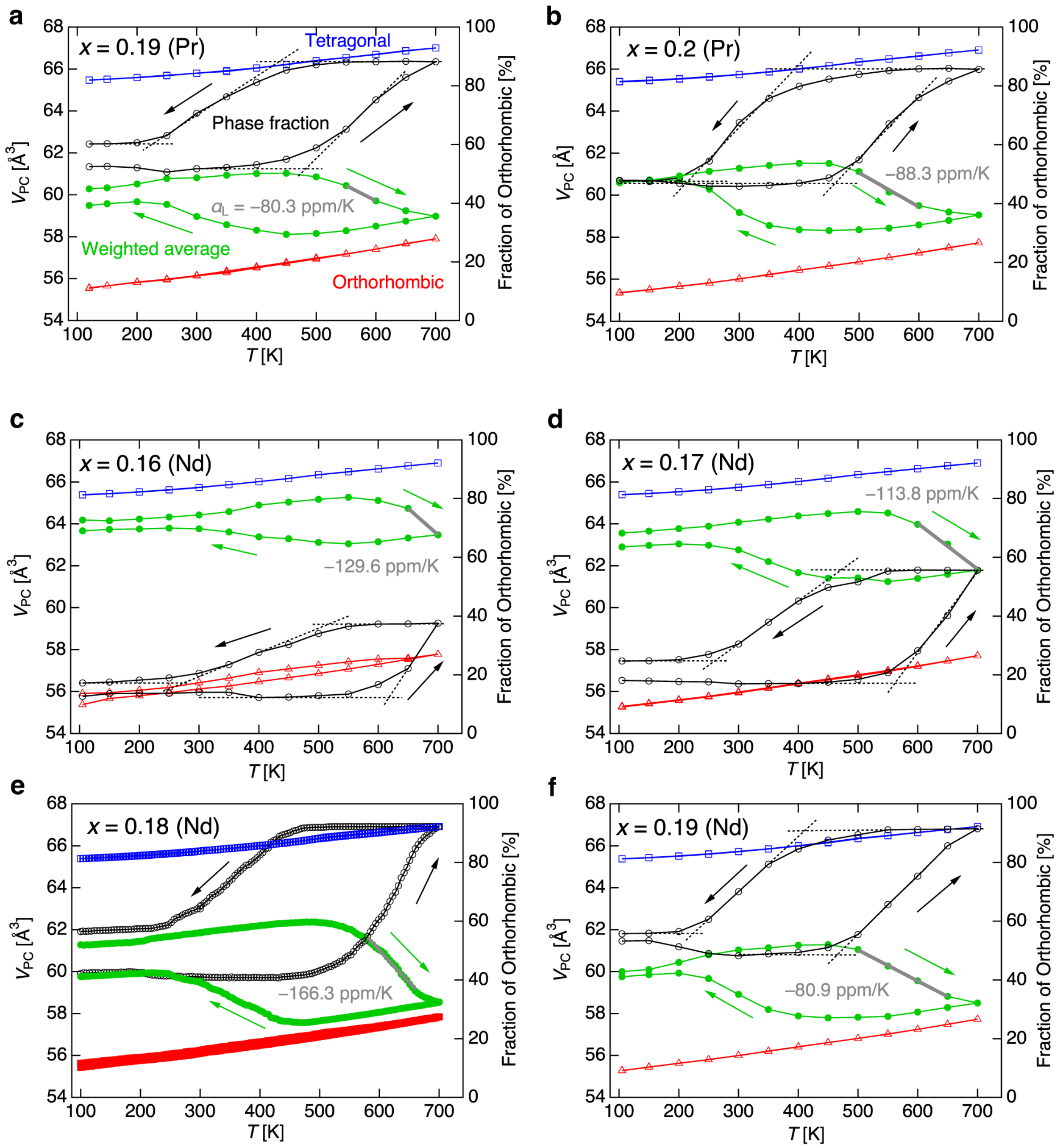

Figure S10. The temperature dependence of pseudo cubic volumes of the tetragonal and orthorhombic phases, the fraction of the orthorhombic phase, and weighted average volume for $Bi_{1-x}Pr_xCoO_3$ (a-b) and $Bi_{1-x}Nd_xCoO_3$ (c-f). Anomalies observed at 300 K are due to the switching of measurement sequences (b, e). Gray bold lines on the weighted average volume indicate results of linear approximation fittings used to calculate the α_Ls.

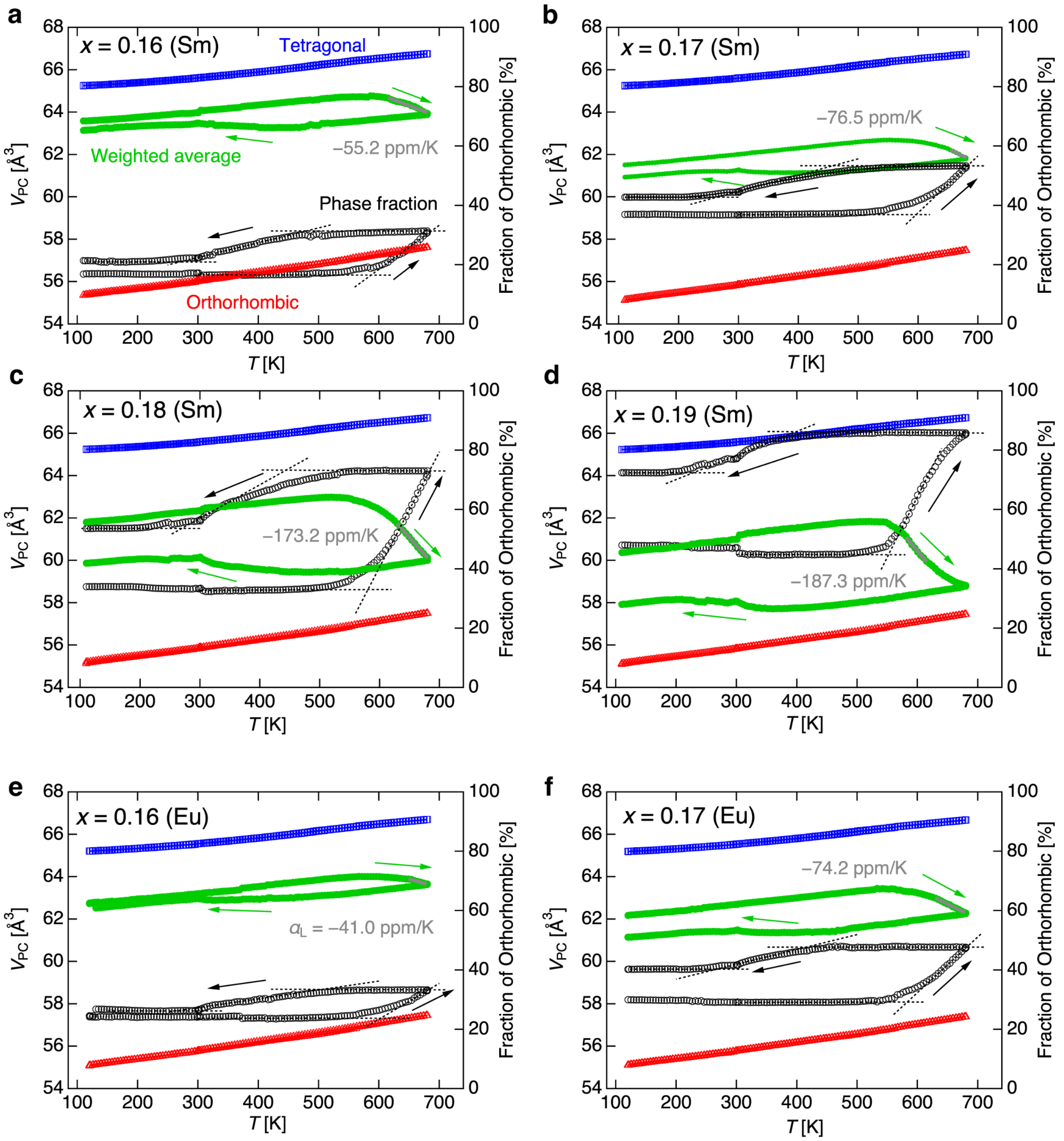

Figure S11. The temperature dependence of pseudo cubic volumes of the tetragonal and orthorhombic phases, the fraction of the orthorhombic phase, and weighted average volume for $Bi_{1-x}Sm_xCoO_3$ (a-d) and $Bi_{1-x}Eu_xCoO_3$ (e-f). Anomalies observed at 300 K are due to the switching of measurement sequences. Gray bold lines on the weighted average volume indicate results of linear approximation fittings used to calculate the α_Ls.

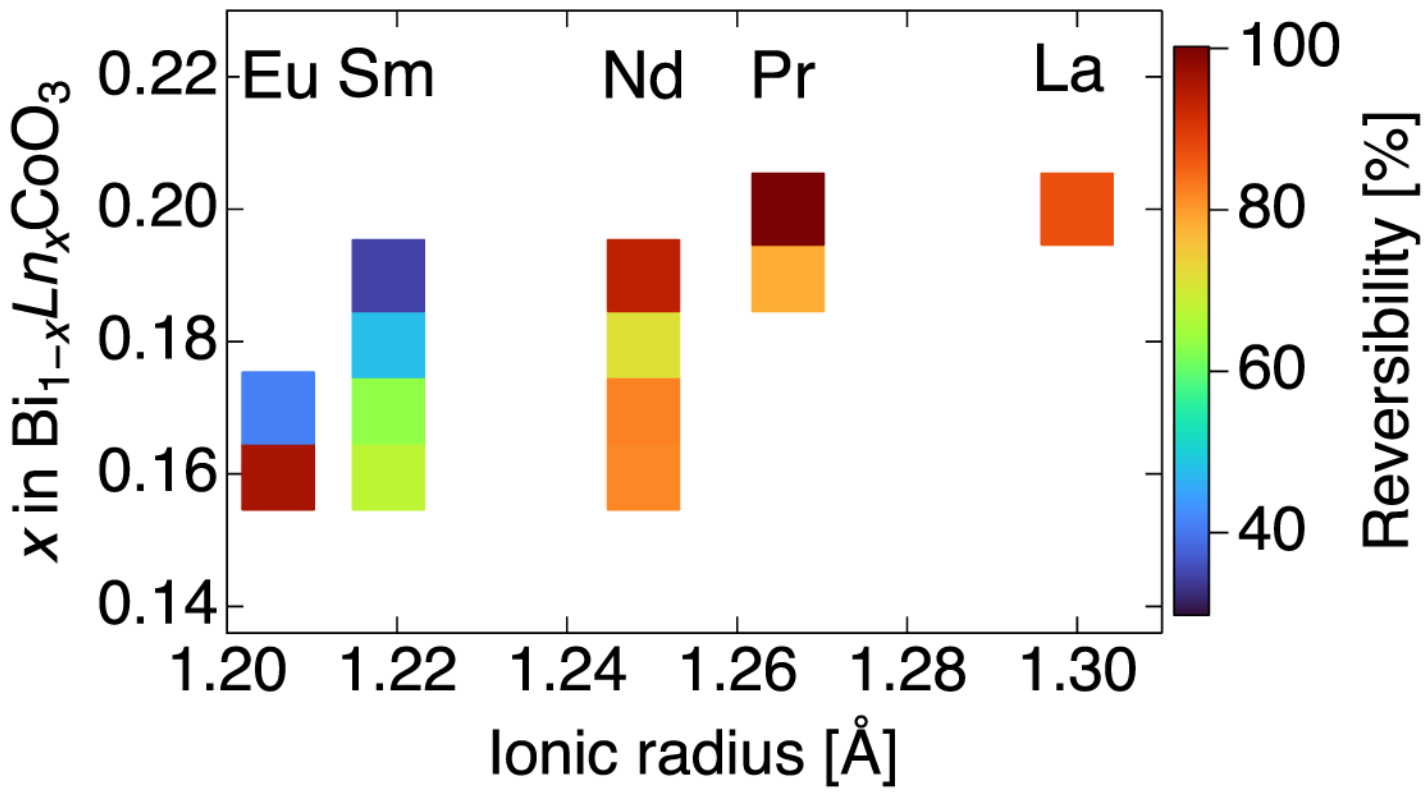

Figure S12. The reversibility of the orthorhombic phase fraction for $Bi_{1-x}Ln_xCoO_3$ as a function of ionic radius and concentration of substituted *Ln* ions. The reversibility is defined as $R = (f_O(T_{Max}) - f'_O(T_{Min})) / (f_O(T_{Max}) - f_O(T_{Min}))$, where $f_O(T_{Max})$ is the fractions of the orthorhombic phase at maximum temperature and $f_O(T_{Min})$ and $f'_O(T_{Min})$ are those at minimum temperature before and after a heat cycle, respectively.

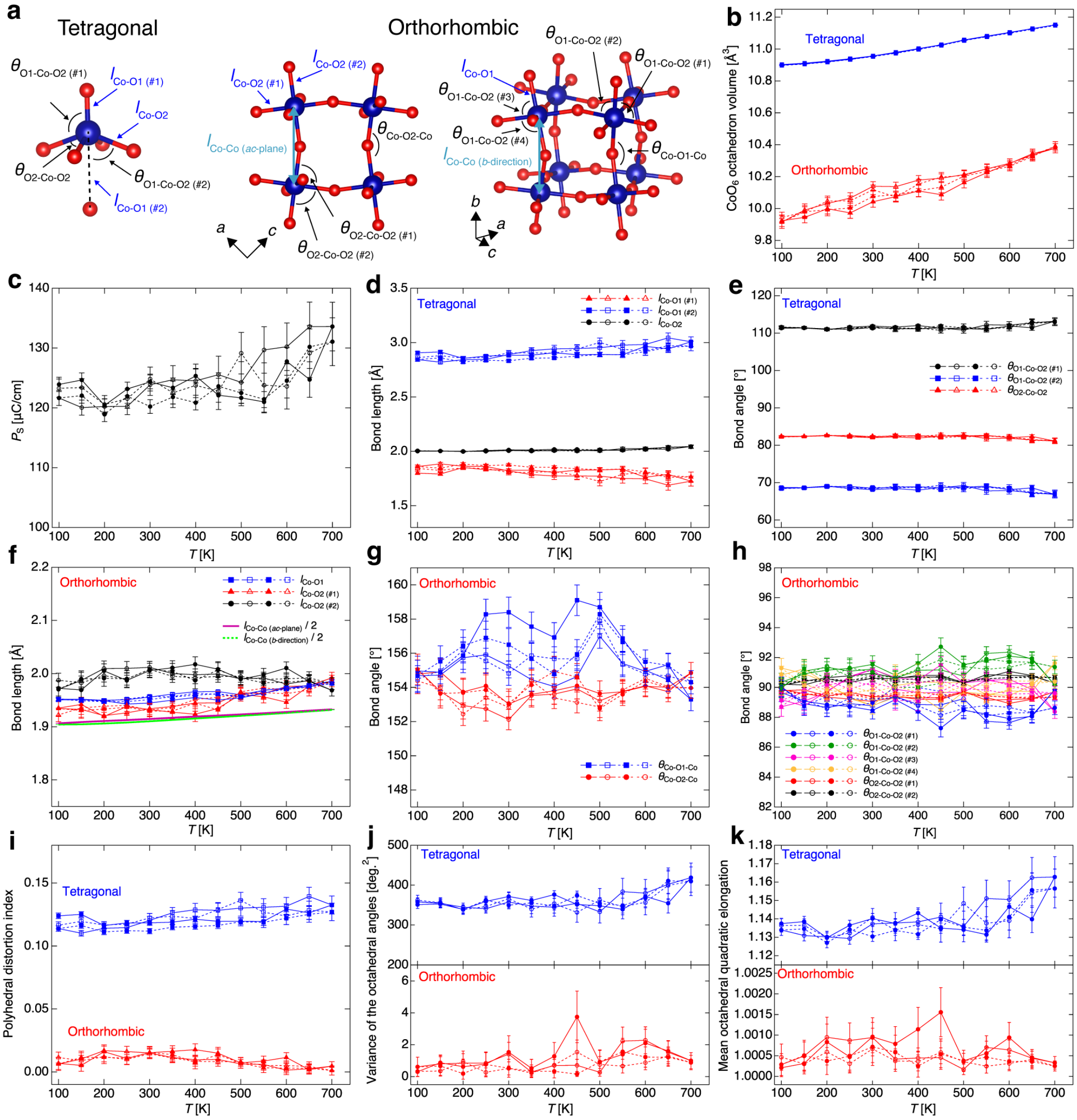

Figure S13. (a) Schematic image of the Co-O bond lengths and the Co-O-Co and O-Co-O bond angles in the tetragonal and orthorhombic phases. Blue and red spheres represent Co and O atoms, respectively. (b-k) The temperature dependence of the CoO_6 octahedron volumes of the tetragonal and orthorhombic phases (b), the spontaneous ferroelectric polarization (P_S) in the tetragonal phase calculated by the point charge model (c), Co-O bond lengths in the tetragonal phase (d), O-Co-O bond angles in the tetragonal phase (e), Co-O bond lengths in the orthorhombic phase (f), O-Co-O bond angles in the orthorhombic phase (i), Co-O-Co bond angles in the orthorhombic phase (h), and the polyhedral distortion index D (g), the variance of the octahedral angles σ^2 (j), and mean octahedral quadratic elongation $\langle\lambda\rangle$ (k) in the tetragonal and orthorhombic phases for $Bi_{0.8}Pr_{0.2}CoO_3$. Solid symbols with solid lines, open symbols with solid lines, solid symbols with dotted lines, open symbols with dotted lines represent data from the 1st heating process, 1st cooling process, 2nd heating process, and 2nd cooling process, respectively.
